# Environment-specific virocell metabolic reprogramming

**DOI:** 10.1093/ismejo/wrae055

**Published:** 2024-03-29

**Authors:** Cristina Howard-Varona, Morgan M Lindback, Jane D Fudyma, Azriel Krongauz, Natalie E Solonenko, Ahmed A Zayed, William B Andreopoulos, Heather M Olson, Young-Mo Kim, Jennifer E Kyle, Tijana Glavina del Rio, Joshua N Adkins, Malak M Tfaily, Subhadeep Paul, Matthew B Sullivan, Melissa B Duhaime

**Affiliations:** Department of Microbiology, The Ohio State University, 484 W 12th Ave, Columbus, OH 43210, United States; Department of Ecology and Evolutionary Biology, University of Michigan, 1105 North University Ave, Ann Arbor, MI 48109, United States; Department of Environmental Science, University of Arizona, 1177 E 4th St, Tucson, AZ 85719, United States; Present address: Department of Plant Pathology, University of California, Davis, One Shields Avenue, Davis, CA 95616, United States; Department of Statistics, The Ohio State University, 1958 Neil Ave, Columbus, OH 43210, United States; Department of Microbiology, The Ohio State University, 484 W 12th Ave, Columbus, OH 43210, United States; Department of Microbiology, The Ohio State University, 484 W 12th Ave, Columbus, OH 43210, United States; US Department of Energy Joint Genome Institute, 1 Cyclotron Road, Berkeley, CA 94720, United States; Present address: Department of Computer Science, San Jose State University, One Washington Square, San Jose CA 95192, United States; Biological Sciences Division, Pacific Northwest National Laboratory, 902 Battelle Blvd, Richland, WA 99354, United States; Biological Sciences Division, Pacific Northwest National Laboratory, 902 Battelle Blvd, Richland, WA 99354, United States; Biological Sciences Division, Pacific Northwest National Laboratory, 902 Battelle Blvd, Richland, WA 99354, United States; US Department of Energy Joint Genome Institute, 1 Cyclotron Road, Berkeley, CA 94720, United States; Biological Sciences Division, Pacific Northwest National Laboratory, 902 Battelle Blvd, Richland, WA 99354, United States; Department of Biomedical Engineering, Oregon Health and Science University, Portland, OR 97239, United States; Department of Environmental Science, University of Arizona, 1177 E 4th St, Tucson, AZ 85719, United States; Department of Statistics, The Ohio State University, 1958 Neil Ave, Columbus, OH 43210, United States; Department of Microbiology, The Ohio State University, 484 W 12th Ave, Columbus, OH 43210, United States; Department of Civil, Environmental and Geodetic Engineering, The Ohio State University, 2070 Neil Ave, Columbus, OH 43210, United States; Center for RNA Biology and Center of Microbiome Science, The Ohio State University, 484 W. 12th Ave, Columbus, OH 43210, United States; Department of Ecology and Evolutionary Biology, University of Michigan, 1105 North University Ave, Ann Arbor, MI 48109, United States

**Keywords:** virus, bacteria, virocell, multi-omics, ecology, marine, metabolic reprogramming, nutrient limitation

## Abstract

Viruses impact microbial systems through killing hosts, horizontal gene transfer, and altering cellular metabolism, consequently impacting nutrient cycles. A virus-infected cell, a “virocell,” is distinct from its uninfected sister cell as the virus commandeers cellular machinery to produce viruses rather than replicate cells. Problematically, virocell responses to the nutrient-limited conditions that abound in nature are poorly understood. Here we used a systems biology approach to investigate virocell metabolic reprogramming under nutrient limitation. Using transcriptomics, proteomics, lipidomics, and endo- and exo-metabolomics, we assessed how low phosphate (low-P) conditions impacted virocells of a marine *Pseudoalteromonas* host when independently infected by two unrelated phages (HP1 and HS2). With the combined stresses of infection and nutrient limitation, a set of nested responses were observed. First, low-P imposed common cellular responses on all cells (virocells and uninfected cells), including activating the canonical P-stress response, and decreasing transcription, translation, and extracellular organic matter consumption. Second, low-P imposed infection-specific responses (for both virocells), including enhancing nitrogen assimilation and fatty acid degradation, and decreasing extracellular lipid relative abundance. Third, low-P suggested virocell-specific strategies. Specifically, HS2-virocells regulated gene expression by increasing transcription and ribosomal protein production, whereas HP1-virocells accumulated host proteins, decreased extracellular peptide relative abundance, and invested in broader energy and resource acquisition. These results suggest that although environmental conditions shape metabolism in common ways regardless of infection, virocell-specific strategies exist to support viral replication during nutrient limitation, and a framework now exists for identifying metabolic strategies of nutrient-limited virocells in nature.

## Graphical Abstract

**Graphical Abstract:**
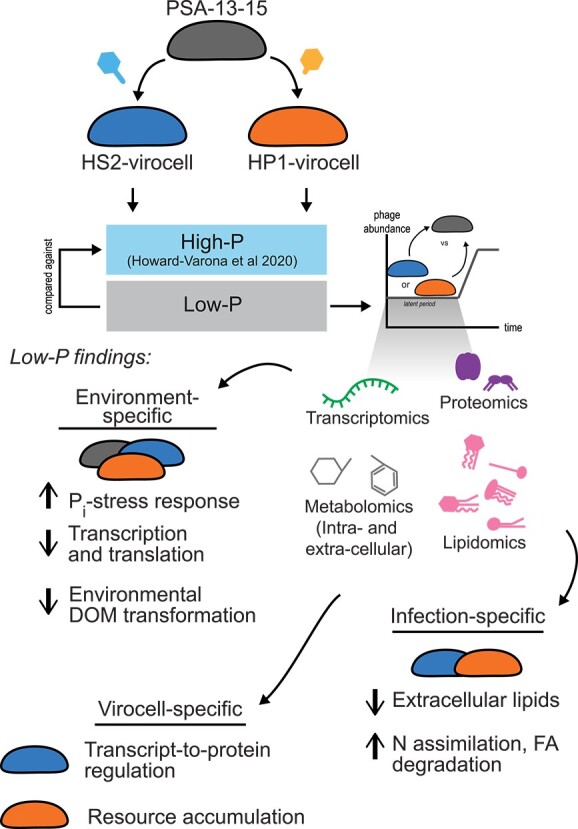

## Introduction

Microbes are central to ecosystem health and function, and microbial diversity and abundance is shaped by bottom-up (nutrients) and top-down (predation) forces. Among predators, viruses influence host mortality, metabolism, and horizontal gene transfer [1–3]. During infection, viruses transform their microbial hosts into new entities termed “virocells” [4] that are metabolically and ecologically distinct from uninfected cells [5–8]. Specifically, in virocells, host machinery is redirected toward obtaining energy and intra- and extra-cellular resources for viral infection [6, 9] in ways that can alter cells and the environment. However, most virocell studies are done in nutrient-replete conditions that do not represent the resource limitation that virocells likely encounter in nature, e.g. the generally low and fluctuating oceanic nutrient levels [10–13]. This leaves the interplay between bottom-up and top-down processes largely unstudied.

Among nutrients, phosphate (P) is essential for diverse biomolecules (e.g. DNA, RNA, modified-proteins, ATP) and is disproportionately higher in viruses than hosts [14]. In the oceans, P is often co-limiting with nitrogen [11, 13, 15, 16] and shapes both viral gene content [17, 18] and infection mechanisms [9, 19–21]. During P-starved *Prochlorococcus* infections by a phage encoding P-acquisition auxiliary metabolic genes, host and phage P-acquisition marker genes were expressed [22], orchestrated by the host’s two-component regulatory system, phoR/phoB [17]. In the unicellular eukaryotic algae *Micromonas pusilla*, transcriptional responses to low-P were stronger than to viral infection [23]. Although these studies show that P-status strongly influences transcripts during infections, little is known about how P-status impacts other biomolecules or virocell metabolic reprogramming in other taxa, nor how P-stressed virocells interact with the environment.

Here we studied the effects of low-P on two *Pseudoalteromonas* virocells obtained from independent infections with dsDNA phages, siphovirus HS2 and podovirus HP1. We follow the host and both phages via time-resolved multi-omics (transcriptomics, proteomics, lipidomics, and endo- and exo-metabolomics) to compare low-P responses (current study) against our prior findings in high-P conditions [7] that are augmented here with lipidomics and metabolomics. Our prior work revealed that metabolic reprogramming was phage-specific in nutrient-rich conditions and likely driven by phage-host genomic complementarity [7]. This current study asks: what are the intra- and extra-cellular impacts of low-P on cells and virocells across integrated omics datatypes?

## Materials and methods

### Data availability

All genomes are available at the Joint Genome Institute’s portal for Integrated Microbial Genomes/Virus (IMG/VR) and GenBank. The IMG submission IDs are 100976 (*Pseudoalteromonas* sp. 13-15), 44764 (PSA-HS2), and 279897 (PSA-HP1). The GenBank IDs are FSRF00000000 (*Pseudoalteromonas* sp. 13-15), KF302036.1 (PSA-HS2), and NC_048630 (PSA-HP1). All cultures are available through the National Collection of Industrial, Food and Marine Bacteria (NCIMB) collection. The NCIMB IDs are NCIMB15513 (*Pseudoalteromonas* sp. 13-15), NCIMB15514 (PSA-HP1), and NCIMB15515 (PSA-HS2). The genome of *Pseudoalteromonas* sp. strain 13-15 was downloaded from NCBI (www.ncbi.nlm.nih.gov) on 20 March 2018. Supplementary datasets are in Zenodo (https://zenodo.org/records/10355633).

### Bacterial growth and phage infections

Growth and infections were conducted as described previously [7, 24, 25] with modifications for phosphate (P) limitation (low-P herein). Briefly, *Pseudoalteromonas* sp. 13-15 were grown shaking at 150 rpm at 21°C in either 1% Z + CNP medium for high-P or 1% Z + CN medium for low-P conditions. These media consisted of 1% Zobell (26 g sea salts, 1 g yeast extract, and 5 g proteose peptone per L), 8.3 mM ammonium sulfate, 0.15 mM phosphoric acid to high-P only, and 11 mM glucose added after autoclaving. The high-P medium had 55 μM of total organic phosphate (TOP) and 48 μM of inorganic phosphate (PO_4_), whereas the low-P medium had 5 μM TOP and 0.6 μM PO_4_ (Supplementary Table S1).

For growth curves, one colony was inoculated into 10 mL of either high-P or low-P media and grown for 12 h while measuring optical density at 600 nm (OD_600_). This starter culture was transferred 1:20 in triplicate into 200 mL of the same medium in 1 L flasks, from which colony forming units (CFUs) and OD_600_ were sampled to obtain OD–CFU regression equations used to estimate cell density for phage infections. Growth rate (h^−1^), calculated during exponential growth as ln(OD_2_/OD_1_)/(time_2_-time_1_), was obtained using R’s “growthcurver” package [26]. Growth was also assessed in this manner in the 1% Zobell medium only (without added C, N, or P).

For phage infections, 5 × 10^8^ cells from the starter culture were transferred to 200 mL in 1 L flasks and grown to mid- to late-exponential phase. Then, ~1 × 10^8^ cells were transferred in triplicate to a 1.5 mL tube and the volume was adjusted to 1 mL. Phages (either PSA-HP1 or PSA-HS2) were then added at a multiplicity of infection (MOI) of 0.1 for initial characterizations, or at MOI ~3 for the time-resolved ‘omics experiments. During infections, phages were allowed to adsorb for 15 min, and then they were diluted 100-fold in a 250 mL flask, or for 10-fold for time-resolved ‘omics sampling, in a 1 L bottle with the same type of medium used for infections, following previously described marine phage-host transcriptomics [7, 27, 28]. Cells, free phages, and total phages were sampled periodically. Cells were spread on Zobell plates and incubated for 2 days at room temperature (RT). Free phages had cells removed with 0.2 μm syringe filters. Free and total phages were enumerated by top-agar plating technique (plaque assay; [29] previously used [7, 24]). Briefly, phages were diluted in 26 psu artificial seawater (26 g sea salts per L), mixed with 0.4 mL of bacterial overnight culture grown in Zobell and 3.5 mL molten soft agar (Zobell medium containing 0.6% low melting point agarose), and dispersed on 20% Zobell agar plates. Plates were incubated at RT and plaques were visible after 1–2 days.

A detailed account of all ‘omics measurements and statistical analyses is provided in the Supplementary Materials and summarized below.

### Transcriptomics

From diluted samples, 15–25 mL were collected in biological triplicates from 0, 30, 60, 80, and 100 min (low-P HP1-virocells and uninfected) or 0, 30, 60, 80, 100, 120, and 140 min (low-P HS2-virocells and uninfected), and pelleted for 11 min at 20000 *g*. The supernatant was discarded before flash-freezing in liquid N_2_. RNA was extracted using the Zymo Quick RNA Mini kit (R1054). RNA concentration and integrity were assessed using the Agilent 2100 Bioanalyzer RNA 6000 Pico assay with the prokaryote protocol. Glow cell sequencing was performed on the HiSeq2500 sequencer using HiSeq TruSeq SBS sequencing kits, v4, following a 2 × 100 indexed run (Illumina, San Diego, CA). Raw gene counts were generated with FeatureCounts whereby both reads from a pair-end run were aligned to the same feature in the reference genome. Read normalization and differential expression (DE) analyses were performed following previously published scripts and procedures [7, 27, 28]. DE analyses were performed between host-infected and uninfected samples at every time point and under the same medium using the statistical package edgeR [30] whereby genes with a false discovery rate and *P* values < 0.05 were considered DE. Metabolic pathway reconstruction was performed with KEGG [31] and Ecocyc [32].

### Proteomics

From diluted samples, 80 mL were collected in biological triplicates from 0, 20, 40, 60, 80, and 100 min (low-P samples) and pelleted for 8–11 min at 20000 *g*. The supernatant was discarded prior to flash-freezing with liquid N_2_. After protein extraction (see supplementary methods), mass-spectrometry (MS) analysis was performed using a Q-Exactive Plus mass spectrometer (Thermo Scientific, San Jose, CA) outfitted with a home-made nano-electrospray ionization interface. MS-GF+ version v2017.01.13 was used to identify peptides from the LC–MS/MS spectra. Search parameters included a +/−20 ppm parent mass tolerance, partial trypsin rules, and dynamic oxidized methionine residues. The candidate protein list was assembled by combining the *Pseudoalteromonas* sp. 13-15 proteome with the 6-frame translation of both phages and a collection of 195 contaminant proteins. The spectra were filtered with an MSGF E-value score of <1 × 10^−9^ and 2 or more unique peptide sequences were required to consider a protein identified. Passing spectra were counted and used as a relative abundance value for comparing across datasets.

Proteomics data are available at MassIVE and the ProteomeXchange repositories with accession numbers MSV000083626 and PXD013204, respectively.

### Lipidomics

From the diluted samples, 40–80 mL from biological triplicates were spun down 8–12 min at 20000 *g* from 0, 15, 30, 40, and 50 min (high-P samples) or 0, 20, 40, 60, and 80 min (low-P samples), then washed 1× with PBS by spinning down 5 min at 20000 *g,* and flash-freezing prior to processing. For the extractions of intracellular lipids and metabolites, MPLEx extraction was used (see Supplementary Methods). Lipid identifications were made using LIQUID [33].

### Endometabolomics

From the diluted samples, 80–90 mL from biological triplicates from 0, 10, 20, 30, and 40 min (high-P samples) or 0, 20, 40, 60, 80, and 100 min (low-P samples) was deposited by vacuum filtration onto a 47 mm 0.4 μm polycarbonate filter and washed with an equal volume of PBS. Three media-only blanks were included. The extraction was the same as for the lipid samples described above. Analyses were done as reported previously [34]. MS data files were processed using Metabolite Detector [35]. Peaks were matched to PNNL augmented version of Agilent metabolomics database and additionally cross-checked with Wiley Registry 11th Edition and NIST17 GC–MS spectral databases. Identified metabolites were validated manually. Peak area values of detected metabolites were log-transformed for further analyses.

### Exometabolomics

From the diluted samples, 90 mL collected at 0, 30, and 60 min in triplicates for all samples were spun down 5000 *g* for 5 min to pellet cells, the supernatant was filtered through a 0.2 μm filter, and flash-frozen. Three respective media-only blanks were included. After removing salts (see supplementary methods), high resolution mass spectra of the filtrate and media-only blanks were collected by direct injection using a Bruker 9.4-Tesla Fourier transform ion cyclotron resonance mass spectrometer (University of Arizona). Putative chemical formulas were assigned using Formultitude (previously named Formularity) software [36]. Gibbs free energy (GFE) was calculated as described previously [37], with peaks that were assigned a putative molecular formula in all samples. Organic matter (OM) transformation analysis for each individual sample replicate was done via network analysis using MetaNetter [38].

### Linear mixed effects models

Separate linear mixed effect models were fitted for host proteins, host transcripts, phage proteins, phage transcripts, phage structural proteins, endometabolites, lipidomic data, and exometabolomic data. These models include a random intercept term to account for longitudinal correlation for genes. Gaussian family distribution is used for the transcript data (FPKM counts). Poisson family distribution is used for protein data (MS counts). Count was predicted by the main effects which are infection status, media type, and time, plus interaction terms between each pair of predictor variables. Type II Wald chi-square test ANOVA was performed to determine whether each term is statistically significant in predicting counts and the relative amount of variance explained by each term.

## Results and discussion

### Overview of infection, transcription, and translation

**Figure 1:**
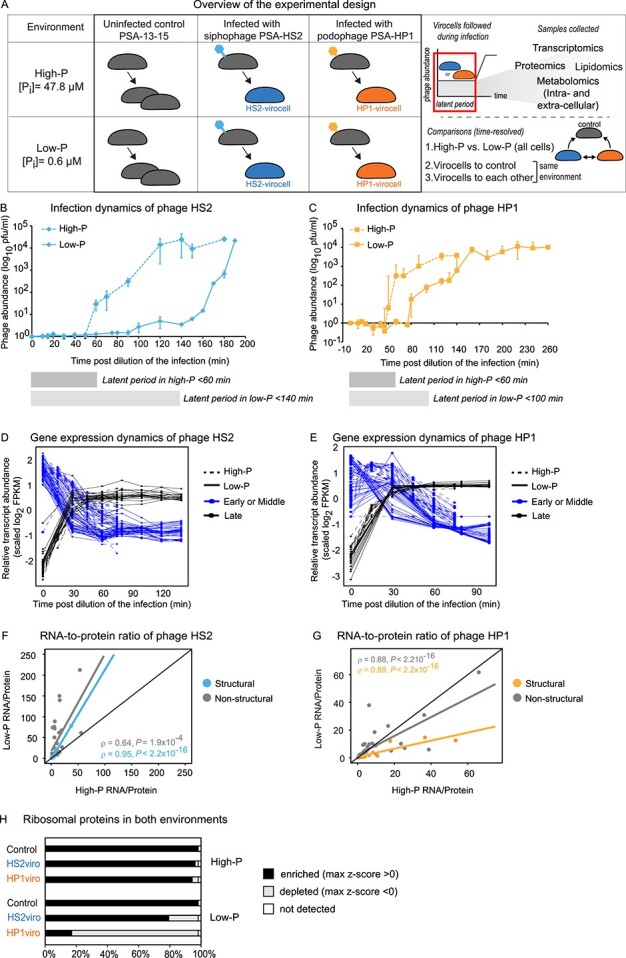
**Experimental design, phage properties, and host** RPs. (**A**) Experimental design. *Pseudoalteromonas* (strain PSA 13-15) was independently infected with two different phages (PSA-HS2 and PSA-HP1) and sampled throughout the latent period ( boxed) for time-resolved multi-omics measurements in either low-P or high-P conditions and compared with uninfected control cells in the same condition. Transcriptomics, proteomics, and exo-metabolomics were all used as primary data, and endometabolomics and lipidomics as supporting data. The manuscript presents the story from three angles: (1) environment-specific effects (high-P versus low-P), (2) infection-specific effects (infected versus uninfected cells), and (3) phage-specific effects (HS2-virocells versus HP1-virocells) in the same environment. (**B**) Infection dynamics of phage PSA-HS2 in both media. (**C**) Infection dynamics of phage PSA-HP1 in both media. For both, (B) and (C), phage abundance, measured as plaque-forming units (pfus) per mL for free phages are represented over time, and 0 min represents 15 min after diluting the infection. The average of three biological replicates is plotted along with the error. All data points have been normalized against the first time point (0 min). Estimates of latent period are represented with rectangle boxes under each graph. (**D**) Gene expression dynamics of phage PSA-HS2 in both media. (**E**) Gene expression dynamics of phage PSA-HP1 in both media. For both, (D) and (E), relative transcript abundance for all genes is plotted as standardized log_2_FPKM (using all time points and media) over infection time, in high-P (dashed) and low-P (solid) media and colored according to earlier (blue) or later (black) temporal expression. (**F**, **G**) Scatter plot of the RNA-to-protein ratio (RNA/protein) for PSA-HS2 (F) and PSA-HP1 (G) phage genes in high-P (x-axis) and low-P (y-axis) conditions. Phage structural genes were determined based on annotation and are colored blue (HS2) or yellow (HP1); non-structural genes displayed in gray. The RNA/protein was calculated by dividing the average normalized transcript abundance (FPKM) by the average normalized protein abundance, both averaged across three replicates. (**H**) Bar plot representing the fraction of RPs that are enriched (z-score > 0), depleted (z-score < 0), or not detected, in every treatment and condition, including the uninfected control, HS2-virocells (HS2viro), and HP1-virocells (HP1viro), obtained from proteomics. Ribosome recycling proteins are not included.

Uninfected cells, HP1-virocells, and HS2-virocells were grown in low phosphate (low-P herein), profiled for multi-omics (transcriptomics, proteomics, endometabolomics, exometabolomics, and lipidomics), and compared with our previously published high-P data [7]. The high-P medium had 11× more total organic P and 77× more inorganic P (P_i_) than the low-P medium (Supplementary Table S1), and low-P significantly (*P* value < 0.05) impacted the composition of each omics data type (Supplementary Fig. S1; Supplementary Table S2). To evaluate the impact of low-P on virocell metabolic reprogramming and ecosystem footprint, virocell transcriptomes, proteomes, endo-metabolomes, endo-lipidomes, and exo-metabolomes were collected (Fig. 1A). To describe these biomolecular changes, we used the following language: (i) *transcripts* were over-expressed (OE), under-expressed (UE), or not differentially expressed (not DE) when their fold-change was >1, <1, or 1, respectively, in infected relative to the uninfected cells in the same medium and time point; (ii) *proteins* were enriched, depleted, or not different from the mean when their z-scores from comparing peptides levels across all proteins, treatments, replicates, and time points were >0, <0, or =0, respectively, and (iii) *metabolites* and *lipids* were enriched or depleted when their fold-change in infected relative to uninfected under the same condition and time point was >1 or <1, respectively.

### Phages display delayed infection, reduced transcription, and opposite fitness strategies under low-P conditions

As expected when bacteria face nutrient limitation [39, 40], uninfected cells had slower growth in low-P (1% Zobell + CN) than in high-P (1% Zobell +CNP) conditions (growth rates ~0.25 h^−1^ and 0.5 h^−1^, respectively) (Supplementary Fig. S2A–D), and 1% Zobell alone did not support cell growth (Supplementary Fig. S2E and F). Additionally, both phage latent periods were delayed ≥2-fold in low-P versus high-P conditions (Fig. 1B and C).

Phage gene expression dynamics, previously measured in high-P conditions [7], were largely maintained under low-P conditions (Fig. 1D and E; Supplementary Fig. S3A and B), consistent with multiple studies showing phage transcriptional programs are relatively invariant [8, 27, 28, 41, 42]. However, ~60% of phage genes had lower expression levels in low-P than in high-P conditions (Supplementary Fig. S3C and D), suggesting low-P impacted phage transcript levels. Because phage transcript versus protein levels during nutrient limitation has not been explored, we assessed RNA-to-protein ratios (R/P), which in uninfected bacteria is both positively correlated with fitness (i.e. higher R/P leads to higher growth rates) and nutrient-sensitive [43, 44]. Phage R/Ps fell above (for HS2) and below (for HP1) the 1:1 line, for both non-structural (Spearman correlation, rho = 0.95 for HS2 and rho = 0.88 for HP1, *P* value < 0.001) and structural (Spearman correlation, rho = 0.64 for HS2 and rho = 0.89 for HP1, *P* value < 0.001) genes (Fig. 1F and G). This suggests opposing fitness strategies, whereby HS2 makes more RNA per protein than HP1 in low-P.

### Low-P reduces transcription and translation in all cells, with virocell-specific differences

To further evaluate phage fitness strategies, and cell/virocell transcription and translation across environments, we conducted linear mixed effects models (LMEMs) on host and phage transcripts and proteins.

Across environments there were significantly (*P* value < 0.0001) fewer host and phage transcripts and proteins in both virocells and uninfected cells in low-P than in high-P conditions (Supplementary Tables S3 and S4). This suggests environment-specific effects where low-P reduced transcription and translation in cells and virocells.

We applied LMEMs to compare the virocells to each other in low-P (Supplementary Tables S3 and S4). For *host*-derived biomolecules, HS2-virocells had significantly (*P* value < 0.001) more transcripts, but fewer proteins than HP1-virocells. These host biomolecule differences could result from virocell differences in either degradation (i.e. host takeover, where one virocell degrades more transcripts/proteins than the other) or production (i.e. resource allocation, where one virocell has a transcript/protein surplus relative to the other). To help discern between the two, we examined ribosomal proteins (RPs). Uninfected cells and HS2-virocells had ≥79% of RPs enriched, whereas HP1-virocells only 17% (Fig. 1H, Supplementary Fig. S4). Additionally, the ribosome recycling factor protein, which reuses ribosomes for translating different mRNAs [45], was only enriched in HP1-virocells (Supplementary Fig. S4). Although there may also be differences in host takeover, these results suggest differences in resource allocation. HS2-virocells likely invest in new protein synthesis, which is possible due to the high codon and amino acid similarity between HS2 and this host [7]. Contrastingly, HP1 is less well matched with this host’s translational machinery [7] and, instead, HP1-virocells likely recycle ribosomes and proteins–which are energetically expensive [46]–as the strategy for responding to fluctuating environments, similar to nutrient-limited uninfected bacteria [47]. As growth (fitness) is proportional to RPs in uninfected bacteria [48], we posit that increased RP production in HS2-virocells contributes to phage HS2’s greater relative fitness [7].

The LMEMs were next applied to *phage*-derived biomolecules in low-P. This revealed that HS2-virocells had significantly fewer transcripts and proteins than HP1-virocells, but HS2-virocells had a significantly faster modeled rate of protein production than HP1-virocells (both *P* values < 0.001; Supplementary Tables S3 and S4). This suggests that, like host proteins in HS2-virocells, HS2 synthesizes phage proteins faster than HP1, likely due to high codon and amino acid similarity between HS2—host [7].

In summary, low-P reduces transcription and translation in all cells and triggers different virocell strategies for transcript/protein resource allocation.

### Virocell metabolic reprogramming strategies in low-P

Given the environment-dependent and virocell-specific biomolecule differences observed above, we next evaluated virocell metabolic reprogramming in low-P.

#### Proteins and transcripts inform the P status differently

**Figure 2:**
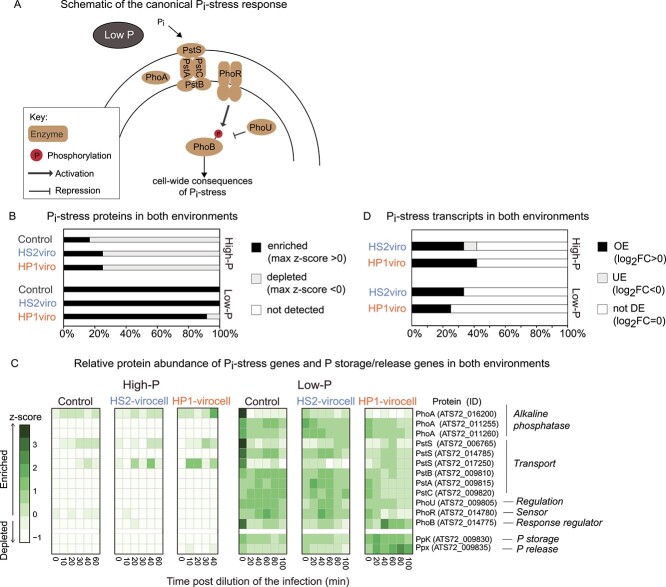
**The canonical inorganic phosphate (P**_**i**_ **)-stress response in high-P and low-P conditions.** (**A**) Cartoon representation of the canonical inorganic phosphate (P_i_)-stress response in a bacterial cell. (**B**) Bar plot representing the fraction of proteins that are enriched (z-score > 0), depleted (z-score < 0), or not detected, in every treatment and condition, obtained from proteomics. (**C**) Heatmap of the relative protein abundance (z-score across all samples and conditions) in high-P and low-P conditions, obtained from proteomics. Proteins are considered enriched if their z-score > 0, and depleted if their z-score < 0. (**D**) Bar plot representing the fraction of genes OE, UE, or not DE in the virocells relative to uninfected control cells in its respective condition, obtained from transcriptomics.

In environments where P_i_ is low, bacteria commonly display the canonical P_i_-stress response, which can be identified by marker genes alkaline phosphatase (*phoA*)*,* phosphate ABC transporter 2C permease protein (*pstA*), phosphate ABC transporter ATP binding protein (*pstB*), phosphate ABC transporter permease (*pstC*)*,* phosphate ABC transporter substrate binding protein (*pstS*), phosphate regulon sensor histidine kinase (*phoR*), and DNA binding response regulator (*phoB*) [49] (Fig. 2A). To ensure that our cells would display the P_i_-stress response, our low-P medium had an order of magnitude less P_i_ than the 0.6 μM P_i_ needed to activate *Escherichia coli*’s P_i_-stress response [50] (Fig. 1A, Supplementary Table S1). We confirmed that low-P uninfected *Pseudoalteromonas* and both virocells displayed the canonical P_i_-stress response because ≥92% of the P_i_-stress proteins were only enriched in low-P and were either depleted or not detected in high-P conditions (Fig. 2B and C; Supplementary Fig. S5A). Then, we examined the transcripts to ask whether infection enhanced or depleted the P_i_-stress response observed in uninfected cells. This revealed that ≤42% of the transcripts were OE in both virocells regardless of the media (Fig. 2D, Supplementary Fig. S5B). These results suggest that phage infection generally elicits a P_i_-stress response even in high-P conditions, which was also observed in cyanovirocells [8], with a relatively small additional P_i_-stress response in low-P conditions.

#### Both low-P virocells favor a specific pathway for nitrogen assimilation

**Figure 3:**
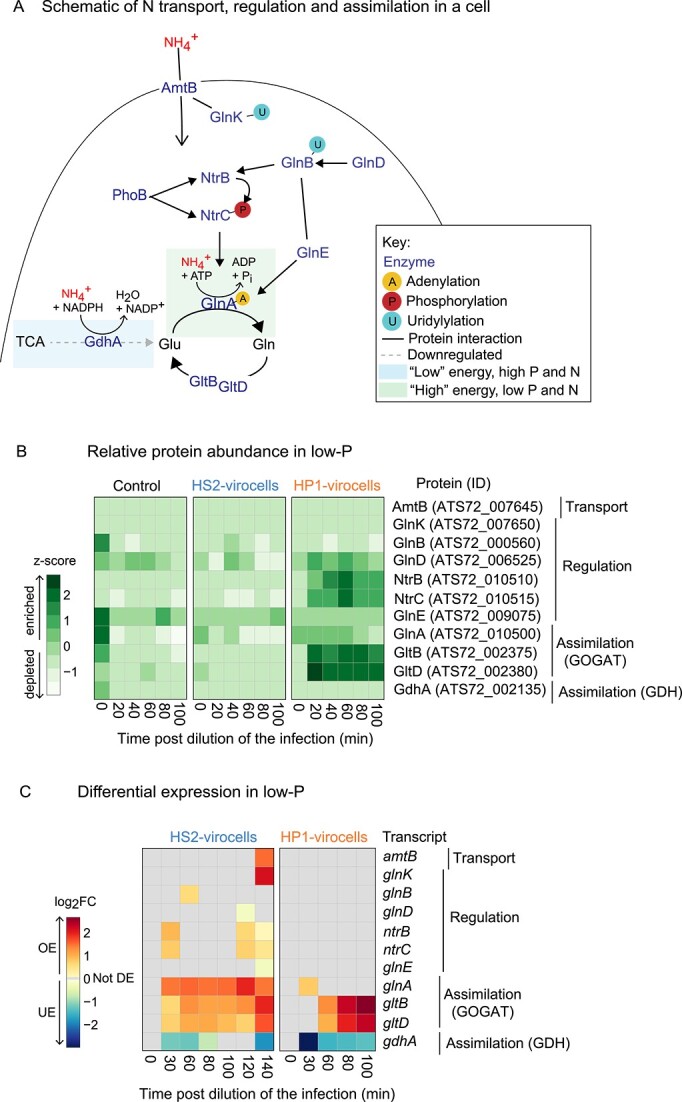
**Nitrogen assimilation during low-P conditions.** (**A**) Cartoon summarizing nitrogen (N) transport, regulation, and assimilation in a cell. (**B**) Heatmap of the relative protein abundance (z-score across all samples and conditions) in low-P, obtained from proteomics. Proteins are considered enriched if their z-score > 0, and depleted if their z-score < 0. (**C**) Heatmap with relative gene expression (log_2_FC relative to uninfected control cells in the same condition and time point) of the N-metabolism genes (N transport, N regulation, and N assimilation) in low-P, obtained from transcriptomics. Genes are OE if log_2_FC > 0, UE if log_2_FC < 0, and not DE if log_2_FC = 0. The gene, ID, and functional category assignment are the same for both heatmaps.

In bacteria, P_i_-stress impacts nitrogen (N) assimilation, through crosstalk mediated by PhoB [49]. Hypothesizing that both virocells would display this crosstalk, we examined transcripts and proteins from N metabolism in low-P, which include (i) ammonium transport (AmtB), (ii) regulation by the regulatory proteins PII-1 (GlnB) and PII-2 (GlnK), the two-component system (NtrB and NtrC), the uridylyltransferase (GlnD), and glutamine synthetase adenylyltransferase (GlnE), and (iii) assimilation by glutamine synthetase (GlnA), glutamate synthase (GltB and GltD), and glutamate dehydrogenase (GdhA) (Fig. 3A). AmtB was not detected in either virocell, but HS2-virocells did OE the transcript (Fig. 3B and C). AmtB is the protein that cells use for active N import into the cell to capture scarce ammonium [51], and in the oceans has been found highly expressed in low-N environments [52].

Of the six N regulation genes, HS2-virocells enriched fewer proteins than HP1-virocells (1 vs 3, respectively), but OE more genes (4 vs 0, respectively; Fig. 3B and C; Supplementary Fig. S6)—consistent with the global trends described above using LMEM, whereby overall HP1-virocells have more host proteins and HS2-virocells more host transcripts relative to each other. These included the kinase *ntrB* and transcriptional regulator *ntrC* that controls transcription of N assimilation genes, and their expression is regulated by PhoB [49]. These co-regulated genes, *ntrBC,* and *phoB,* were only OE in low-P HS2-virocells (Fig. 3C), suggesting a HS2-virocell-specific transcriptional control of the known crosstalk between P_i_-stress and regulation of N assimilation.

Both virocells favored the glutamine oxoglutarate aminotransferase/glutamate synthase (GOGAT) over the glutamate dehydrogenase (GDH) cycle. Specifically, *glnA* from GOGAT was OE in both virocells and its protein enriched, whereas *gdhA* from GDH was UE and its protein depleted (Fig. 3B and C; Supplementary Fig. S6). In uninfected bacteria, GOGAT is the cell’s ATP-dependent pathway for N assimilation that is preferred when P and N levels are low, but when energy is sufficient; whereas GDH utilizes TCA cycle intermediates in an ATP-independent manner and is preferred when energy is low and P and N levels are high [53] (Fig. 3A). This resource trade-off has not been previously described in virocells, but here it suggests virocells are limited for TCA cycle intermediates in addition to P and N.

### Virocell-specific central carbon metabolism reprogramming toward energy and resource production in low-P

**Figure 4:**
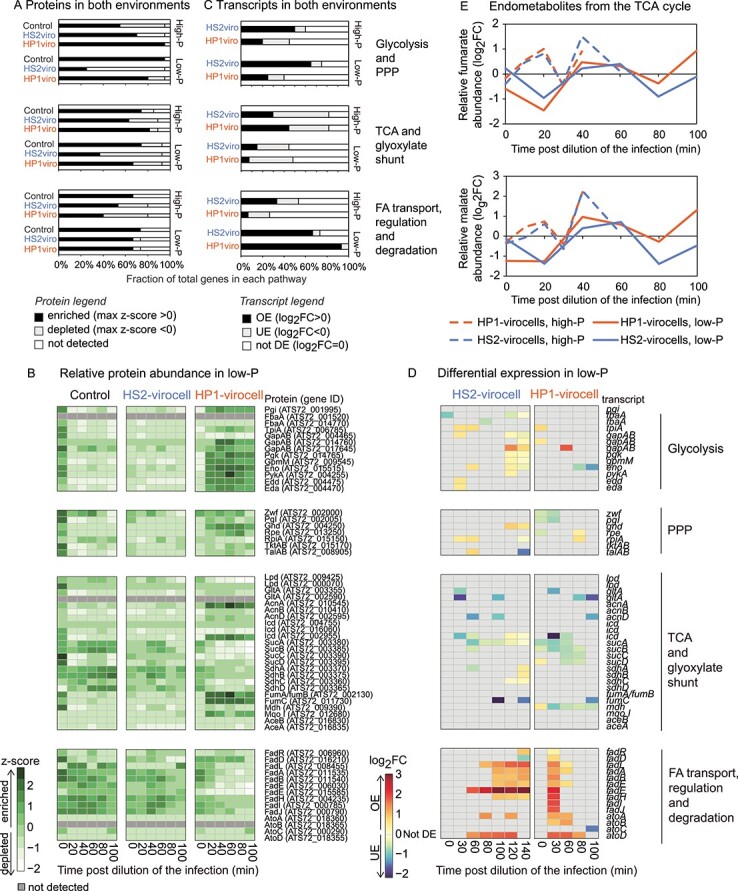
**Energy metabolism in high-P and low-P conditions.** (**A**) Bar plot representing the fraction of proteins that are enriched, depleted, or not detected, in every treatment and condition, obtained from proteomics. (**B**) Heatmap of the relative protein abundance (z-score across all samples and conditions) in low-P, obtained from proteomics. Proteins are considered enriched if their z-score > 0, and depleted if their z-score < 0. Dark gray are proteins that are not detected in our dataset. (**C**) Bar plot representing the fraction of genes OE, UE, or not DE in the virocells relative to uninfected control cells in the respective condition and time point, obtained from transcriptomics. (**D**) Heatmap with relative gene expression (log_2_FC relative to uninfected control cells in the same condition and time point) in low-P conditions, obtained from transcriptomics. Genes are OE if log_2_FC > 0, UE if log_2_FC < 0, and not DE if log_2_FC = 0. The gene, ID, and functional category assignment on the right are the same for both heatmaps. (**E**) Relative abundance of the TCA cycle metabolites detected in the endometabolome, represented as log_2_FC in the virocells relative to uninfected control cells in the same condition and time point. Protein names for B and D: glucose-6-phosphate isomerase (Pgi), fructose-bisphosphate aldolase (FbaA), triose-phosphate isomerase (TpiA), glyceraldehyde-3-phosphate dehydrogenase (GapAB), phosphoglycerate kinase (PgK), phosphoglycerate mutase (GpmM), enolase (Eno), pyruvate kinase (PykA), phosphogluconate dehydratase (Edd), 2-keto-3-deoxygluconate-6-phosphate aldolase (Eda), glucose-6-phosphate dehydrogenase (Zwf), 6-phosphogluconolactonase (Pgl), 6-phosphogluconate dehydrogenase (Gnd), ribulose-phosphate 3-epimerase (Rpe), ribose-5-phosphate isomerase (RpiA), transketolase (TktAB), transaldolase (TalAB), dihydrolipoyl dehydrogenase (Lpd), citrate synthase (GltA), aconitate hydratase (AcnA), aconitate hydratase B (AcnB), 2 methylisocitrate dehydratase (AcnD), isocitrate dehydrogenase (Icd), 2-oxoglutarate dehydrogenase subunit E1 (SucA), dihydrolipoamide succinyltransferase (SucB), succinate CoA ligase subunit beta (SucC), succinate CoA ligase subunit alpha (SucD), succinate dehydrogenase flavoprotein subunit (SdhA), succinate dehydrogenase iron sulfur subunit (SdhB), succinate dehydrogenase 2C cytochrome b556 subunit (SdhC), succinate dehydrogenase 2C hydrophobic membrane anchor protein (SdhD), fumarate hydratase (FumA/FumB), class II fumarate hydratase (FumC), malate dehydrogenase (Mdh), malate:quinone oxidoreductase (Mqo I), isocitrate lyase (AceA), malate synthase (AceB), DNA-binding transcriptional dual regulator (FadR), long-chain-fatty-acid—CoA ligase (FadD), long-chain fatty acid outer membrane channel (FadL), 3-ketoacyl-CoA thiolase (FadA), enoyl-CoA hydratase (FadB), acyl-CoA dehydrogenase (FadE), 2,4-dienoyl-CoA reductase (FadH), 3-ketoacyl-CoA thiolase (FadI), 3-hydroxyacyl-CoA dehydrogenase (FadJ), beta complex (AtoA), acetyl-CoA acetyltransferase (AtoB), DNA-binding transcriptional activator (AtoC), alpha complex (AtoD).

As viruses need energy and resources for replication [46], and central carbon metabolism (CCM) is a source of both [54], we asked whether CCM would be impacted by infection in low-P (Supplementary Figs S7 and S8). In low-P relative to high-P conditions, the fraction of enriched proteins increased by 21% in uninfected cells and decreased by 52 and 17% in HS2-virocells and HP1-virocells, respectively (Fig. 4A and B; Supplementary Fig. S8A). This suggests environment-specific effects on infection whereby virocells reduce CCM activity in low-P conditions.

Virocells in low-P differed: HP1-virocells had significantly (*P* value < 0.05) more CCM proteins than HS2-virocells, but OE fewer transcripts (Fig. 4A–D). Additionally, among the CCM pathways, HP1-virocells enriched more proteins from glycolysis, pentose phosphate pathway (PPP), and the tricarboxylic acid (TCA) cycle than HS2-virocells: 77, 86, and 67% (HP1-virocells) versus 15, 43, and 37% (HS2-virocells) for each respective pathway (Fig. 4A). Finally, HP1-virocells had 11% (fumarate) and 30% (malate) higher TCA cycle metabolite fold-change than HS2-virocells in low-P, with maximum fold-change of 2.5 (HP1-virocells) and 1.6 (HS2-virocells) of these metabolites relative to uninfected cells in low-P (Fig. 4E). Together these data suggest virocell-specific effects with higher CCM activity in HP1-virocells than in HS2-virocells in low-P.

To contextualize these findings, prior work revealed that HS2 shares higher genomic similarity with the host than HP1, and that in high-P conditions, HP1-virocells have greater energy and resource demands [7, 24]. Applied to our current findings, it appears that HP1-virocells during nutrient limitation continue to have greater energy and resource needs relative to those of HS2-virocells, which may be alleviated through increasing CCM activity.

### Fatty acid degradation is enhanced in low-P, especially in HP1-virocells

Because energy can also come from fatty acids (FAs) (Supplementary Fig. S7), we asked if cells and virocells were degrading FAs. We found that FA transport, regulation, and degradation proteins were significantly (*P* value < 0.05) more abundant in low-P than in high-P conditions by 9% (uninfected), 20% (HS2-virocells), and 40% (HP1-virocells) (Fig. 4A). Furthermore, in low-P, 67% of those proteins were enriched in both virocells, but fewer transcripts were OE in HS2-virocells (67%) than in HP1-virocells (93%) (Fig. 4A–D, Supplementary Fig. S8). These results suggest that (i) cells enhance FA degradation in response to low-P conditions, regardless of infection, and (ii) low-P HP1-virocells reprogram FA degradation more so than HS2-virocells, presumably due to the greater energy demand.

To test the hypotheses that low-P cells degraded FA, and HP1-virocells did so the most, we characterized the intracellular lipidome. An LMEM revealed that all detected lipids had significantly (*P* value < 0.0001) lower abundance in low-P than in high-P conditions for both virocells, but not for uninfected cells (Supplementary Table S5). Additionally, lipid depletion was phage-specific, as HP1-virocells had significantly (*P* value < 0.0001) fewer lipids than HS2-virocells in low-P (Supplementary Table S5). These data suggest that lipid depletion is environment-, infection- and phage-specific, as both virocells, but not uninfected cells, deplete lipids in response to low-P conditions, and HP1-virocells more so.

### Virocell ecosystem footprint

Viruses impact nutrient availability and cycling, especially carbon, in myriad ways [55–63]. Yet, the extent to which virocells impact extracellular dissolved organic carbon (DOC) composition *during* infection is poorly understood, particularly in contrasting environmental conditions relevant at the micron-scale [64]. To address this unknown, we tracked DOC chemical composition via high resolution MS as a diagnostic of environmental transformation, asking: what are the relative impacts of P availability and infection on cells, virocells, and their ecosystem footprints?

### Low-P has a greater impact on DOC than phage infection

We evaluated which factors contributed to the observed semi-quantitative variation in DOC chemical composition between samples. From a principal component analysis, in which 49.7% of the observed variation was captured in the first two principal components (Supplementary Fig. S1E), low-P was the only factor that significantly correlated with the observed variation in the DOC molecule types detected (linear regression, *P* value < 0.001, R^2^ = 0.0004; time, *P* value = 0.06, and infection status, *P* value = 0.39, were not significant). Group-aggregated analyses also indicated that low-P was more significant (ANOVA, *F* = 26.9, *P* value < 0.001) than time (ANOVA, *F* = 2.7, *P* value < 0.005), and infection was not significant (ANOVA, *F* = 0.9, *P* value > 0.05; Supplementary Table S6). Together, these data suggest that, even though marine viruses are known to impact OM [65, 66], low-P conditions more strongly shape DOC chemical composition than phage *during infection*.

### Infection enhances extracellular lipid and peptide utilization in low-P

We investigated the chemical composition of the DOC across all samples and time points. Over 7000 DOC compounds were detected ranging in mass from ~154–1195 Da, with 53.4% not assignable to molecular formulas or biogeochemical classes, and the rest consisting mainly of peptide-, lipid-, and polyphenol-like compounds (**Supplementary materials**).

**Figure 5:**
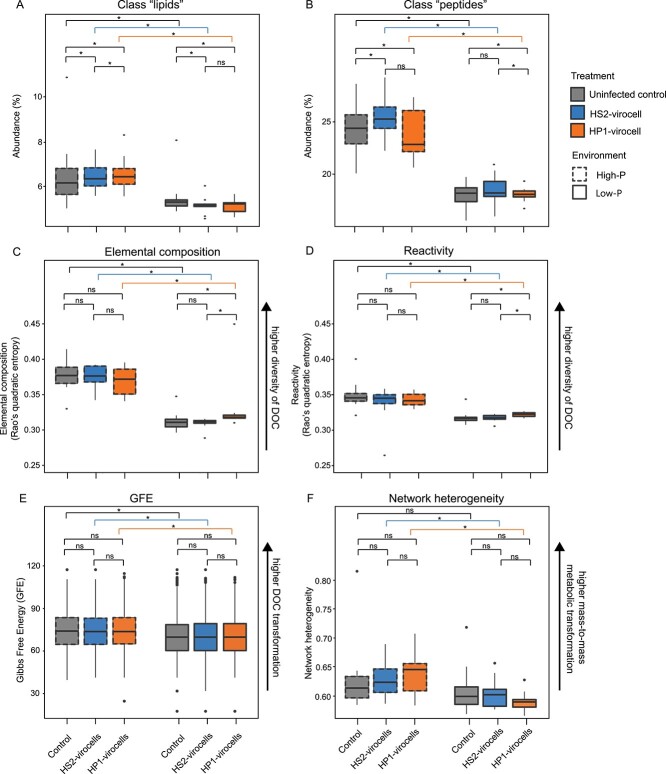
**Ecosystem footprint inferred from** DOC **composition from the exometabolome collected during infection.** Exometabolome samples were collected for virocells and uninfected controls during the latent period, at 0, 30, and 60 min post-dilution of each infection in both environments. (**A** and **B**) Boxplots representing the range of percentages of total exometabolites identified in each treatment that were classified as (A) lipid-like and (B) peptide-like compounds for uninfected cells, HS2-virocells, and HP1-virocells. (**C**) Elemental composition, (**D**) reactivity, (**E**) GFE of exometabolites, and (**F**) network heterogeneity in uninfected control cells, HP1- and HS2-virocells. Asterisks denote the *P* value resulting from a Wilcoxon test comparing virocells to uninfected cells, or between high-P and low-P conditions for each cell (uninfected, HS2-virocell or HP1-virocell), with “^*^” denoting significant comparisons (*P* value < 0.05) and “ns” denoting “not significant” comparisons (*P* value ≥ 0.05).

How did low-P and infection impact these DOC classes (Supplementary Table S6)? First, in low-P versus high-P conditions, both virocells and uninfected cells had significantly (*P* value < 0.05) fewer lipid- and peptide-like compounds (Fig. 5A and B), suggesting environment-specific effects on these exometabolites. Second, in low-P, both HS2-and HP1-virocells had significantly (*P* value < 0.05) fewer lipids than uninfected cells (Fig. 5A), suggesting infection-specific effects on extracellular lipid abundance. However, there were no significant differences (*P* value > 0.05) between virocells to each other (Fig. 5A), suggesting extracellular lipids in low-P were not impacted by phage (i.e. phage-independent). For peptides, HP1-virocells had significantly (*P* value < 0.05) fewer extracellular peptides relative to both uninfected cells and HS2-virocells (Fig. 5B), suggesting phage-specific effects. Coupled with our intracellular observations (Fig. 3B and C; Fig. 4A–E), peptides (for N) and FAs (for energy and P) may have been partially derived extracellularly and contributed to these trends. Extracellular resource sourcing has been observed in other non-nutrient-limited virocells such as from *Synechococcus* [62, 67] and *Sulfitobacter* [55], and likely helps close the elemental composition gap that exists between viruses and their hosts [14].

### DOC transformation decreases in low-P and is virocell-specific

Given ~50% of the DOC species were unclassified, we calculated the functional diversity of the exometabolome using Rao’s quadratic entropy (RQE) [68–70]. RQE measures differences between DOC pools by considering both compound abundances and differences in chemical properties, such as elemental composition and reactivity [37]. This leverages a prior hypothesis that similarities in chemical properties translate to similarities in ecological function [71–74]. First, in low-P versus high-P conditions, all cells had significantly (*P* value < 0.05) lower DOC RQE values, i.e. lower functional diversity, in terms of both elemental composition and reactivity (Fig. 5C and D; Supplementary Table S6), suggesting environment-specific effects. Second, in low-P, only HP1-virocells were significantly different (*P* value < 0.05), to both HS2-virocells and uninfected cells (Fig. 5C and D), suggesting virocell-specific effects whereby HP1-virocells likely transformed or exuded more diverse compounds during infection than the other cells.

Another way of understanding these DOC transformations is through their GFE and network heterogeneity (NH). GFE is routinely used in soil ecology to understand microbial OM transformation, as it represents the maximum work that can be performed in a thermodynamic system [37, 75–77]. NH values denote the degree of substrate and end-product transformations in an entire system [78]. Higher NH and GFE values denote microbial systems that are actively transforming metabolites to more recalcitrant states. First, in low-P versus high-P conditions, all cells had significantly (*P* value < 0.05) lower GFE (Fig. 5E), with no significant differences (*P* value > 0.05) between virocells, nor between virocells and uninfected cells (Fig. 5E; Supplementary Table S6). Furthermore, GFE distribution showed a more diverse range of labile and recalcitrant metabolites in high-P than in low-P conditions (Supplementary Fig. S9), suggesting DOC transformation was environment-specific. Contrastingly, NH was significantly (*P* value < 0.05) lower in low-P than in high-P conditions for both virocells, but not for uninfected cells (*P* value > 0.05; Fig. 5F). Second, in low-P there were no significant differences (*P* value > 0.05) between virocells or between virocells and uninfected cells for either GFE or NH (Fig. 5F, Supplementary Table S6), suggesting an infection-dependent metabolic transformation of OM *due to the environment*. Together, these pre-lysis observations suggest that (i) low-P reduces DOC transformation (i.e. lower GFE) in all cells, and (ii) infection in low-P results in fewer metabolic transformations of DOC (i.e. lower NH), but these transformations did not significantly change the bioavailability of the system.

### Virocell metabolic reprogramming and ecosystem footprint in low-P conditions

**Figure 6:**
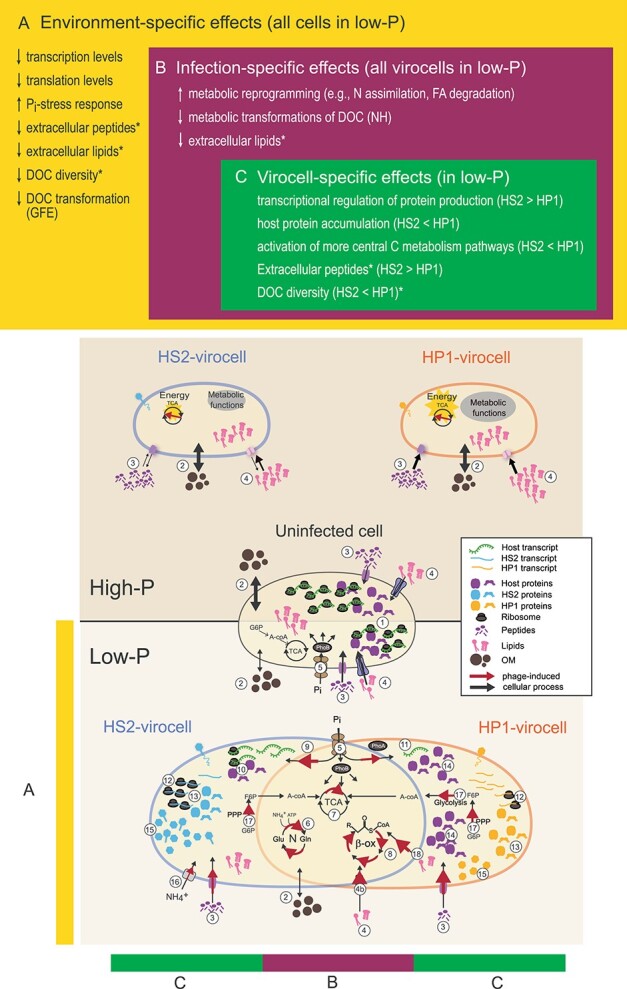
**Virocell metabolic reprogramming and ecosystem footprint of cells and virocells under varying phosphate conditions.** (**A**) Environment-specific effects (common to all cells and virocells as a response to low-P): decrease in (1) intracellular transcripts and proteins, (2) extracellular DOM transformation and diversity, (3) extracellular peptides, (4) extracellular lipids, and (5) the activation of the canonical P_i_-stress response in low-P. Asterisks indicate that extracellular peptides, lipids, and DOC diversity are also affected in B and/or C. (**B**) Infection-specific effects (common to both virocells, but not uninfected control cells, as a response to low-P): (5) enhancing the P_i_-stress response of the uninfected host, (6) assimilating N via the GOGAT pathway, (7) activating central C metabolism, (4b) importing and (8) degrading lipids, which likely results in decreasing the extracellular lipid pool, and fewer metabolic transformations. (**C**) Virocell-specific effects (specific to each phage infection in response to low-P): HS2-virocells (9) invest in P to make more transcripts and, using the host’s translational machinery, (10) rapidly synthesize host proteins when needed. Contrastingly, HP1-virocells struggle to make (11) transcripts and (12) RPs, and are less well-matched with the host translational machinery, thus resulting in (13) slower phage protein translation than HS2-virocells. Therefore, instead of relying on gene expression (transcript-to-protein) as HS2-virocells, HP1-virocell’s strategy may be to (14) accumulate existing host proteins (recycling instead of synthesis) to be able to respond to fluctuating environments. The trade-off, however, is that host proteins occupy precious cellular space needed to make phage proteins, (15) resulting perhaps in fewer HP1 virions compared with HS2. Other virocell-specific phenomena are that (16) HS2-virocells likely use active transport to import ammonium, (3) HP1-virocells have fewer extracellular peptides, and HP1-virocells need more energy and resources, as shown by (17) the activation of a wider range of energy pathways in central C metabolism and (18) the increase in FA degradation relative to HS2-virocells. Cells and virocells are not drawn to scale and molecules do not reflect quantity.

Here we report virocell intracellular metabolic reprogramming–following transcripts, proteins, metabolites, and lipids–and extracellular DOC changes in low-P, relative to previously published high-P conditions assayed via transcripts and proteins only [7]. We find three tiers of nested responses to the dual stresses of low-P and viral infection: (i) shared between all cells regardless of infection (i.e. environment-specific effects, but infection-independent), (ii) shared between virocells (i.e. infection-specific, but virocell-independent effects), and (iii) unique to each virocell due to the infecting phage (i.e. virocell- or phage-specific effects) (Fig. 6).

For *environment-specific* effects, in low-P all cells (uninfected, HP1-virocells, and HS2-virocells) reduced overall transcription and translation, activated the canonical P_i_-stress response, had fewer extracellular peptides and lipids, had a less diverse DOC pool (i.e. lower elemental composition and reactivity), and decreased overall DOC transformation (i.e. lower GFE), relative to high-P conditions (Fig. 6A).

For *infection-specific* effects, both low-P virocells had fewer extracellular lipids than uninfected cells, lower metabolic transformations (i.e. NH) than in high-P conditions, and reprogrammed intracellular metabolic pathways of N assimilation and energy (Fig. 6B).

For *virocell-specific* effects in low-P, extracellularly HP1-virocells had fewer peptides and higher DOC diversity (i.e. RQE) than HS2-virocells (Fig. 6C). Intracellularly, the virocells appeared to adopt different strategies for managing resource needs: gene expression regulation (HS2-virocells) versus resource accumulation and recycling (HP1-virocells). Based on nutrient-limited uninfected bacteria [79], there appears to be a trade-off between transcriptionally regulating proteins to save energy (HS2-virocells) versus recycling proteins (i.e. resource accumulation) to quickly respond to environmental changes (HP1-virocells). For example, HS2-virocells, using similar codons as the host [7], likely maintain low protein abundance and invest in transcriptional regulation for responsiveness (Fig. 6C). Contrastingly, HP1-virocells, being more codon-mismatched with the host, likely require more resources and energy (inferred from [7] and our CCM, and FA degradation results here), produce fewer RPs, recycle existing ribosomes, and maintain high host protein abundance to be metabolically ready. Based on prior work, this would come at the expense of utilizing valuable cellular space or other limiting resources for virions [80]. Therefore, nutrient limitation triggers common virocell responses as well as nuanced metabolic reprogramming strategies imposed by resource and energy needs that may ultimately narrow the nutrient limitation gap and enable phages to reproduce equally well across environments (as seen from the similar phage titers obtained in our closed system, Fig. 1B and C). Finally, we posit that the nature and magnitude of the ecosystem footprint left by a virocell is not static, but rather depends on the environment of such infection.

This study provides a framework for developing empirical datasets needed to bridge the gap between laboratory phage-host experimental multi-omics datasets across taxa and conditions, and those in nature. Beyond experimental measurements, helping empiricists refine which data are most impactful in metabolic models, and myriad advances, such as in genome-scale and flux balance analysis modeling [81], will be critical to place observational data into the mechanistic frameworks needed to predict dynamic outcomes. These steps are critical toward establishing the rules of life that govern the virocells that transform planet Earth [82, 83].

## Supplementary Material

HowardVarona_Lindback_R2_SI_clean_v2_wrae055

TableS1_mediacomposition_rev2_wrae055

TableS2_ANOVA_phage_vs_environment_wrae055

TableS3_Linear_mixed_effects_model_transcripts_rev2_wrae055

TableS4_Linear_mixed_effects_model_proteins_rev2_wrae055

TableS5_Linear_mixed_effects_model_endoLipidomics_rev2_wrae055

TableS6_DOC_stats_rev2_wrae055

## References

[1] Fuhrman JA . Marine viruses and their biogeochemical and ecological effects. Nature 1999;399:541–8. 10.1038/21119 10376593

[2] Wilhelm SW , Suttle CA. Viruses and nutrient cycles in the sea: viruses play critical roles in the structure and function of aquatic food webs. Bioscience 1999;49:781–8. 10.2307/1313569

[3] Suttle CA . Marine viruses–major players in the global ecosystem. Nat Rev Microbiol 2007;5:801–12. 10.1038/nrmicro1750 17853907

[4] Forterre P . The virocell concept and environmental microbiology. ISME J 2013;7:233–6. 10.1038/ismej.2012.110 23038175 PMC3554396

[5] Rosenwasser S , Ziv C, Creveld SG et al. Virocell metabolism: metabolic innovations during host-virus interactions in the ocean. Trends Microbiol 2016;24:821–32. 10.1016/j.tim.2016.06.006 27395772

[6] Zimmerman AE , Howard-Varona C, Needham DM et al. Metabolic and biogeochemical consequences of viral infection in aquatic ecosystems. Nat Rev Microbiol 2019;18:21–34. 10.1038/s41579-019-0270-x 31690825

[7] Howard-Varona C , Lindback MM, Bastien GE et al. Phage-specific metabolic reprogramming of virocells. ISME J. 2020;14:881–95. 10.1038/s41396-019-0580-z 31896786 PMC7082346

[8] Howard-Varona C , Roux S, Bowen BP et al. Protist impacts on marine cyanovirocell metabolism. ISME Commun 2022;2:1–14. 10.1038/s43705-022-00169-6 37938263 PMC9723779

[9] Warwick-Dugdale J , Buchholz HH, Allen MJ et al. Host-hijacking and planktonic piracy: how phages command the microbial high seas. Virol J 2019;16:15. 10.1186/s12985-019-1120-1 30709355 PMC6359870

[10] Flombaum P , Gallegos JL, Gordillo RA et al. Present and future global distributions of the marine *Cyanobacteria Prochlorococcus* and *Synechococcus*. Proc Natl Acad Sci USA 2013;110:9824–9. 10.1073/pnas.1307701110 23703908 PMC3683724

[11] Martiny AC , Lomas MW, Fu W et al. Biogeochemical controls of surface ocean phosphate. Sci Adv 2019;5:1–10. 10.1126/sciadv.aax0341 PMC6713502 31489372

[12] Tanioka T , Garcia CA, Larkin AA et al. Global patterns and predictors of C:N:P in marine ecosystems. Commun Earth Environ 2022;3:271. 10.1038/s43247-022-00603-6 36407846 PMC9640808

[13] Moore CM , Mills MM, Arrigo KR et al. Processes and patterns of oceanic nutrient limitation. Nat Geosci 2013;6:701–10. 10.1038/ngeo1765

[14] Jover LF , Effler TC, Buchan A et al. The elemental composition of virus particles: implications for marine biogeochemical cycles. Nat Rev Micro 2014;12:519–28. 10.1038/nrmicro3289 24931044

[15] Wu J , Sunda W, Boyle EA et al. Phosphate depletion in the western North Atlantic Ocean. Science 2000;289:759–62. 10.1126/science.289.5480.759 10926534

[16] Mills MM , Ridame C, Davey M et al. Iron and phosphorus co-limit nitrogen fixation in the eastern tropical North Atlantic. Nature 2004;429:292–4. 10.1038/nature02550 15152251

[17] Zeng Q , Chisholm SW. Marine viruses exploit their host’s two-component regulatory system in response to resource limitation. Curr Biol 2012;22:124–8. 10.1016/j.cub.2011.11.055 22244998

[18] Kelly L , Ding H, Huang KH et al. Genetic diversity in cultured and wild marine cyanomyoviruses reveals phosphorus stress as a strong selective agent. ISME J. 2013;7:1827–41. 10.1038/ismej.2013.58 23657361 PMC3749497

[19] Wilson WH , Carr NG, Mann NH. The effect of phosphate status on the kinetics of cyanophage infection in the oceanic cyanobacterium *Synechococcus* sp. WH78031. J Phycol 1996;32:506–16. 10.1111/j.0022-3646.1996.00506.x

[20] Monier A , Welsh RM, Gentemann C et al. Phosphate transporters in marine phytoplankton and their viruses: cross-domain commonalities in viral-host gene exchanges. Environ Microbiol 2012;14:162–76. 10.1111/j.1462-2920.2011.02576.x 21914098 PMC3429862

[21] Maat DS , Crawfurd KJ, Timmermans KR et al. Elevated CO2 and phosphate limitation favor *Micromonas pusilla* through stimulated growth and reduced viral impact. Appl Environ Microbiol 2014;80:3119–27. 10.1128/AEM.03639-13 24610859 PMC4018922

[22] Lin X , Ding H, Zeng Q. Transcriptomic response during phage infection of a marine cyanobacterium under phosphorus-limited conditions. Environ Microbiol 2016;18:450–60. 10.1111/1462-2920.13104 26522011

[23] Bachy C , Charlesworth CJ, Chan AM et al. Transcriptional responses of the marine green alga *Micromonas pusilla* and an infecting prasinovirus under different phosphate conditions. Environ Microbiol 2018;20:2898–912. 10.1111/1462-2920.14273 29749714

[24] Duhaime MB , Solonenko N, Roux S et al. Comparative omics and trait analyses of marine *Pseudoalteromonas* phages advance the phage OTU concept. Front Microbiol 2017;8:1241. 10.3389/fmicb.2017.01241 28729861 PMC5498523

[25] Holmfeldt K , Howard-Varona C, Solonenko N et al. Contrasting genomic patterns and infection strategies of two co-existing *Bacteroidetes* podovirus genera. Environ Microbiol 2014;16:2501–13. 10.1111/1462-2920.12391 24428166

[26] Sprouffske K , Wagner A. Growthcurver: an R package for obtaining interpretable metrics from microbial growth curves. BMC Bioinformatics 2016;17:172. 10.1186/s12859-016-1016-7 27094401 PMC4837600

[27] Howard-Varona C , Roux S, Dore H et al. Regulation of infection efficiency in a globally abundant marine *Bacteriodetes* virus. ISME J 2017;11:284–95. 10.1038/ismej.2016.81 27187794 PMC5335546

[28] Howard-Varona C , Hargreaves KR, Solonenko NE et al. Multiple mechanisms drive phage infection efficiency in nearly identical hosts. ISME J. 2018;12:1605–18. 10.1038/s41396-018-0099-8 29568113 PMC5955906

[29] Sambrook J , Fritsch EF, Maniatis T et al. Molecular Cloning: A Laboratory Manual. Cold Spring Harbor, NY, USA: Cold Spring Harbor Laboratory Press, 1989.

[30] Robinson MD , McCarthy DJ, Smyth GK. edgeR: a Bioconductor package for differential expression analysis of digital gene expression data. Bioinformatics 2010;26:139–40. 10.1093/bioinformatics/btp616 19910308 PMC2796818

[31] Kanehisa M , Goto S. KEGG: Kyoto encyclopedia of genes and genomes. Nucleic Acids Res 2000;28:27–30. 10.1093/nar/28.1.27 10592173 PMC102409

[32] Keseler IM , Collado-Vides J, Santos-Zavaleta A et al. EcoCyc: a comprehensive database of *Escherichia coli* biology. Nucleic Acids Res 2011;39:D583–90. 10.1093/nar/gkq1143 21097882 PMC3013716

[33] Kyle JE , Crowell KL, Casey CP et al. LIQUID: an-open source software for identifying lipids in LC-MS/MS-based lipidomics data. Bioinforma Oxf Engl 2017;33:1744–6. 10.1093/bioinformatics/btx046 PMC5860051 28158427

[34] Snijders AM , Langley SA, Kim YM et al. Influence of early life exposure, host genetics and diet on the mouse gut microbiome and metabolome. Nat Microbiol 2016;2:1–8. 10.1038/nmicrobiol.2016.221 27892936

[35] Hiller K , Hangebrauk J, Jäger C et al. MetaboliteDetector: comprehensive analysis tool for targeted and nontargeted GC/MS based metabolome analysis. Anal Chem 2009;81:3429–39. 10.1021/ac802689c 19358599

[36] Tolić N , Liu Y, Liyu A et al. Formularity: software for automated formula assignment of natural and other organic matter from ultrahigh-resolution mass spectra. Anal Chem 2017;89:12659–65. 10.1021/acs.analchem.7b03318 29120613

[37] Ayala-Ortiz C , Graf-Grachet N, Freire-Zapata V et al. MetaboDirect: an analytical pipeline for the processing of FT-ICR MS-based metabolomic data. Microbiome 2023;11:28. 10.1186/s40168-023-01476-3 36803638 PMC9936664

[38] Burgess KEV , Borutzki Y, Rankin N et al. MetaNetter 2: a Cytoscape plugin for ab initio network analysis and metabolite feature classification. J Chromatogr B Analyt Technol Biomed Life Sci 2017;1071:68–74. 10.1016/j.jchromb.2017.08.015 PMC5726607 29030098

[39] Monod J . The growth of bacterial cultures. Ann Rev Microbiol 1949;3:371–94. 10.1146/annurev.mi.03.100149.002103

[40] Merchant SS , Helmann JD. Elemental economy: microbial strategies for optimizing growth in the face of nutrient limitation. Adv Microb Physiol 2012;60:91–210 22633059 10.1016/B978-0-12-398264-3.00002-4 PMC4100946

[41] Doron S , Fedida A, Hernndez-Prieto MA et al. Transcriptome dynamics of a broad host-range cyanophage and its hosts. ISME J. 2016;10:1437–55. 10.1038/ismej.2015.210 26623542 PMC5029184

[42] Nilsson E , Li K, Hoetzinger M et al. Nutrient driven transcriptional changes during phage infection in an aquatic *Gammaproteobacterium*. Environ Microbiol 2022;24:2270–81. 10.1111/1462-2920.15904 35049095 PMC9305737

[43] Li SHJ , Li Z, Park JO et al. *Escherichia coli* translation strategies differ across carbon, nitrogen and phosphorus limitation conditions. Nat Microbiol 2018;3:939–47. 10.1038/s41564-018-0199-2 30038306 PMC6278830

[44] Klumpp S , Hwa T. Bacterial growth: global effects on gene expression, growth feedback and proteome partition. Curr Opin Biotechnol 2014;28:96–102. 10.1016/j.copbio.2014.01.001 24495512 PMC4111964

[45] Kiel MC , Kaji H, Kaji A. Ribosome recycling: an essential process of protein synthesis. Biochem Mol Biol Educ 2007;35:40–4. 10.1002/bmb.6 21591054

[46] Mahmoudabadi G , Milo R, Phillips R. Energetic cost of building a virus. Proc Natl Acad Sci USA 2017;114:E4324–33. 10.1073/pnas.1701670114 28512219 PMC5465929

[47] Mori M , Schink S, Erickson DW et al. Quantifying the benefit of a proteome reserve in fluctuating environments. Nat Commun 2017;8:1225. 10.1038/s41467-017-01242-8 29089487 PMC5663898

[48] Tollerson R , Ibba M. Translational regulation of environmental adaptation in bacteria. J Biol Chem 2020;295:10434–45. 10.1074/jbc.REV120.012742 32518156 PMC7383399

[49] Santos-Beneit F . The Pho regulon: a huge regulatory network in bacteria. Front Microbiol 2015;6:1–13. 10.3389/fmicb.2015.00402 25983732 PMC4415409

[50] Marzan LW , Shimizu K. Metabolic regulation of *Escherichia coli* and its *phoB* and *phoR* genes knockout mutants under phosphate and nitrogen limitations as well as at acidic condition. Microb Cell Factories 2011;10:39. 10.1186/1475-2859-10-39 PMC3129296 21599905

[51] Shimizu K . Regulation systems of bacteria such as *Escherichia coli* in response to nutrient limitation and environmental stresses. Meta 2014;4:1–35. 10.3390/metabo4010001 PMC4018673 24958385

[52] Coles VJ , Stukel MR, Brooks MT et al. Ocean biogeochemistry modeled with emergent trait-based genomics. Science 2017;358:1149–54. 10.1126/science.aan5712 29191900

[53] van Heeswijk WC , Westerhoff HV, Boogerd FC. Nitrogen assimilation in *Escherichia coli*: putting molecular data into a systems perspective. Microbiol Mol Biol Rev 2013;77:628–95. 10.1128/MMBR.00025-13 24296575 PMC3973380

[54] Noor E , Eden E, Milo R et al. Central carbon metabolism as a minimal biochemical walk between precursors for biomass and energy. Mol Cell 2010;39:809–20. 10.1016/j.molcel.2010.08.031 20832731

[55] Ankrah NYD , May AL, Middleton JL et al. Phage infection of an environmentally relevant marine bacterium alters host metabolism and lysate composition. ISME J 2013;8:1089–100. 10.1038/ismej.2013.216 24304672 PMC3996693

[56] Kuhlisch C , Schleyer G, Shahaf N et al. Viral infection of algal blooms leaves a unique metabolic footprint on the dissolved organic matter in the ocean. Sci Adv 2021;7:4680–98. 10.1126/sciadv.abf4680 PMC8213229 34144983

[57] Middelboe M , Jørgensen NOG. Viral lysis of bacteria: an important source of dissolved amino acids and cell wall compounds. J Mar Biol Assoc U K 2006;86:605–12. 10.1017/S0025315406013518

[58] Lønborg C , Middelboe M, Brussaard CPD. Viral lysis of *Micromonas pusilla*: impacts on dissolved organic matter production and composition. Biogeochemistry 2013;116:231–40. 10.1007/s10533-013-9853-1

[59] Gobler CJ , Hutchins DA, Fisher NS et al. Release and bioavailability of C, N, P Se, and Fe following viral lysis of a marine chrysophyte. Limnol Oceanogr 1997;42:1492–504. 10.4319/lo.1997.42.7.1492

[60] Zhang C , Dang H, Azam F et al. Evolving paradigms in biological carbon cycling in the ocean. Natl Sci Rev 2018;5:481–99. 10.1093/nsr/nwy074

[61] Zhang R , Wei W, Cai L. The fate and biogeochemical cycling of viral elements. Nat Rev Micro 2014;12:850–1. 10.1038/nrmicro3384 25396723

[62] Ma X , Coleman ML, Waldbauer JR. Distinct molecular signatures in dissolved organic matter produced by viral lysis of marine cyanobacteria. Environ Microbiol 2018;20:3001–11. 10.1111/1462-2920.14338 30047191

[63] Zhao Z , Gonsior M, Schmitt-Kopplin P et al. Microbial transformation of virus-induced dissolved organic matter from picocyanobacteria: coupling of bacterial diversity and DOM chemodiversity. ISME J. 2019;13:2551–65. 10.1038/s41396-019-0449-1 31227815 PMC6776026

[64] Stocker R . Marine microbes see a sea of gradients. Science 2012;338:628–33. 10.1126/science.1208929 23118182

[65] Weitz JS , Wilhelm SW. Ocean viruses and their effects on microbial communities and biogeochemical cycles. F1000 Biol Rep 2012;4:17. 22991582 10.3410/B4-17 PMC3434959

[66] Worden AZ , Follows MJ, Giovannoni SJ et al. Environmental science. Rethinking the marine carbon cycle: factoring in the multifarious lifestyles of microbes. Science 2015;347:1257594. 10.1126/science.1257594 25678667

[67] Waldbauer JR , Coleman ML, Rizzo AI et al. Nitrogen sourcing during viral infection of marine cyanobacteria. Proc Natl Acad Sci USA 2019;116:15590–5. 10.1073/pnas.1901856116 31308237 PMC6681717

[68] Rao CR . Diversity and dissimilarity coefficients: a unified approach. Theor Popul Biol 1982;21:24–43. 10.1016/0040-5809(82)90004-1

[69] Botta-Dukát Z . Rao’s quadratic entropy as a measure of functional diversity based on multiple traits. J Veg Sci 2005;16:533–40. 10.1111/j.1654-1103.2005.tb02393.x

[70] Laliberte E , Legendre P. A distance-based framework for measuring functional diversity from multiple traits. Ecology 2010;91:299–305. 10.1890/08-2244.1 20380219

[71] Danczak RE , Chu RK, Fansler SJ et al. Using metacommunity ecology to understand environmental metabolomes. Nat Commun 2020;11:6369. 10.1038/s41467-020-19989-y 33311510 PMC7732844

[72] Mentges A , Feenders C, Seibt M et al. Functional molecular diversity of marine dissolved organic matter is reduced during degradation. Front Mar Sci 2017;4:194. 10.3389/fmars.2017.00194

[73] Landa M , Cottrell MT, Kirchman DL et al. Phylogenetic and structural response of heterotrophic bacteria to dissolved organic matter of different chemical composition in a continuous culture study. Environ Microbiol 2014;16:1668–81. 10.1111/1462-2920.12242 24020678

[74] Sun L , Perdue EM, Meyer JL et al. Use of elemental composition to predict bioavailability of dissolved organic matter in a Georgia river. Limnol Oceanogr 1997;42:714–21. 10.4319/lo.1997.42.4.0714

[75] AminiTabrizi R , Wilson RM, Fudyma JD et al. Controls on soil organic matter degradation and subsequent greenhouse gas emissions across a permafrost thaw gradient in northern Sweden. Front Earth Sci 2020;8:381. 10.3389/feart.2020.557961

[76] Zhang J , Feng Y, Wu M et al. Evaluation of microbe-driven soil organic matter quantity and quality by thermodynamic theory. mBio 12:e03252–20. 33622716 10.1128/mBio.03252-20 PMC8545108

[77] LaRowe DE , Van Cappellen P. Degradation of natural organic matter: a thermodynamic analysis. Geochim Cosmochim Acta 2011;75:2030–42. 10.1016/j.gca.2011.01.020

[78] Longnecker K , Kujawinski EB. Using network analysis to discern compositional patterns in ultrahigh-resolution mass spectrometry data of dissolved organic matter. Rapid Commun Mass Spectrom RCM 2016;30:2388–94. 10.1002/rcm.7719 27524402

[79] Wessely F , Bartl M, Guthke R et al. Optimal regulatory strategies for metabolic pathways in *Escherichia coli* depending on protein costs. Mol Syst Biol 2011;7:515. 10.1038/msb.2011.46 21772263 PMC3159982

[80] Weitz JS . Quantitative Viral Ecology. Princeton, NJ: Princeton University Press, 2016, The future of quantitative viral ecology

[81] Fang X , Lloyd CJ, Palsson BO. Reconstructing organisms in silico: genome-scale models and their emerging applications. Nat Rev Microbiol 2020;18:731–43. 10.1038/s41579-020-00440-4 32958892 PMC7981288

[82] Howard-Varona C , Hargreaves KR, Abedon ST et al. Lysogeny in nature: mechanisms, impact and ecology of temperate phages. ISME J. 2017;11:1511–20. 10.1038/ismej.2017.16 28291233 PMC5520141

[83] Correa AMS , Howard-Varona C, Coy SR et al. Revisiting the rules of life for viruses of microorganisms. Nat Rev Microbiol. 2021;19:501–13. 10.1038/s41579-021-00530-x 33762712

