# Supplementary material for: Environment-specific virocell metabolic reprogramming
Source: ISME J. 2024 Mar 29;18(1):wrae055. doi: 10.1093/ismejo/wrae055 (PMC11170926; doi:10.1093/ismejo/wrae055)
Supplement: HowardVarona_Lindback_R2_SI_clean_v2_wrae055 [file howardvarona_lindback_r2_si_clean_v2_wrae055.docx]

**Environment-specific virocell metabolic reprogramming**

**SUPPLEMENTARY MATERIAL**

# **Contents**

1. Supplementary methods
2. Figure S1
3. Figure S2
4. Figure S3
5. Figure S4
6. Figure S5
7. Figure S6
8. Figure S7
9. Figure S8
10. Figure S9
11. Figure S10
12. Figure S11
13. Figure S12
14. Figure S13
15. Table S1
16. Table S2
17. Table S3
18. Table S4
19. Table S5
20. Table S6
21. Supplementary references

# **Supplementary methods:**

## Scripts are from our previous work (1) and can be found in Cyverse (<http://datacommons.cyverse.org/browse/iplant/home/shared/iVirus/Pseudoalteromonas_Omics>).

## Genome-wide transcriptomics:

All accession numbers for the raw data deposited onto NCBI can be found in Zenodo (<https://zenodo.org/records/10355633>).

With the extracted RNA, the optional in-column DNase I treatment was used. Alterations to the manufacturer’s protocol included a 10 min room temperature (RT) incubation with lysis buffer in step “I.”, omission of step “II.1.”, and a 1 min RT incubation with water in step “III.7”. Ribo-Zero was used for removing rRNA and libraries were prepared with TruSeq Stranded Total RNA HT with total RNA starting material of 100 ng per sample and 10 cycles of Polymerase Chain Reaction (PCR) for library amplification. Libraries were quantified using KAPA Biosystem’s next-generation sequencing library quantitative-PCR kit and run on a Roche LightCycler 480 real-time PCR instrument. The quantified libraries were then multiplexed with other libraries and together prepared for sequencing on the Illumina HiSeq sequencing platform utilizing a TruSeq paired-end cluster kit, v4, and Illumina’s cBot instrument to generate a clustered flow cell for sequencing. Glow cell sequencing was performed on the Illumina HiSeq2500 sequencer using HiSeq TruSeq SBS sequencing kits, v4, following a 2x100 indexed run recipe.

Plate-based RNA sample preparation was performed on the PerkinElmer Sciclone NGS robotic liquid handling system using Illumina’s Ribo-Zero rRNA Removal Kit (Bacteria) and the TruSeq Stranded Total RNA HT sample preparation kit following the protocol outlined by Illumina in their user guide:<http://support.illumina.com/sequencing/sequencing_kits/truseq_stranded_total_rna_ht_sample_prep_kit.htm>, and with the following conditions: total RNA starting material of 100 ng per sample and 10 cycles of Polymerase Chain Reaction (PCR) for library amplification. Sequencing generated 57 libraries from low-P conditions (see Zenodo), adding to the 54 already available from the high-P study (1), whereby reads were mapped to both contigs of the bacterial genome and phages. The genbank phage files were converted to fasta using Artemis and the gff file was produced with the bioperl script bp_genbank2gff3 (<https://github.com/bioperl/bioperl-live/blob/master/scripts/Bio-DB-GFF/bp_genbank2gff3.pl>).

Raw gene counts were generated with FeatureCounts whereby both reads from a pair-end run were aligned to the same feature in the reference genome. Read normalization and differential expression analyses were performed following previously published scripts and procedures (1–4).

Filtering, trimming and contaminant-removal was with BBDuk (<http://jgi.doe.gov/data-and-tools/bb-tools/>) and BBMap (<https://sourceforge.net/projects/bbmap/>). BBMap was also used to align the filtered reads to the reference genome, allowing only unique mappings. Reads were mapped to genes, pseudogenes and riboswitch features from their gff files. For the host, the product information was manually added to genes by linking gene and Protein Homology features on the basis of their start-end coordinates. This gave the product= tag for each start-end coordinate, which was inserted after the gene features. As the riboswitches did not have "product=" in the original gff, we manually set "product=riboswitch". Additionally, the riboswitch locus_tags were renamed to prefix them with the start and end coordinates. The phage CDS features were used for mapping.

Generated reads were trimmed with BBDuk to remove adapter sequence and where quality dropped to 0. Raw reads were evaluated for artifact sequence by kmer matching (kmer=25), allowing 1 mismatch. Detected artifacts were trimmed from the 3' end of the reads. RNA spike-in reads and PhiX reads were also removed, and so were reads that contained 1 or more 'N' bases, had an average quality score across the read less than 10 or had a minimum length <= 51 bp or 33% of the full read length. BBMap-mapped reads to masked human, cat, dog and mouse references at 93% identity were removed. Reads aligned to common microbial contaminants, or ribosomal rRNA, were also removed.

For the low-P dataset, median coverage to the host genome was 489x, to phage HS2 it was 5423x, and to phage HP1 it was 27516x.

Read counts of each phage and host were normalized separately using the R software package *edge*R in Bioconductor (5). The resulting matrices were used for calculating the FPKM (Fragments per kilobase per million mapped reads) values (6) for all genes and for differential expression (DE) analyses.

DE analyses were performed between host-infected and uninfected samples at every time point and under the same growth medium using *edgeR* (5) whereby genes with a false discovery rate (FDR) and *P value*s<0.05 were considered DE. To compare the two different phage infections, FPKM values from infected cells were normalized as previously described (1–3) such that FPKM_i_ =*a* × FPKM*_x_*+(1−*a*) × FPKM_c_, where FPKM_i_ includes the total RNA-seq signal obtained from ‘infected' samples (which includes infected and uninfected cells), FPKM*_x_* is the expression of just the infected cells (and what needs to be solved for in the previous equation), *a* is the fraction of infected cells and FPKM*_c_* is the expression of non-infected (control) cells. Heatmaps were generated with the Pheatmap R package. The entire procedure can be found in an in-house R-script on Cyverse (<http://datacommons.cyverse.org/browse/iplant/home/shared/iVirus/Pseudoalteromonas_Omics>).

## Genome-wide proteomics:

Proteomes had low quantity and required filter-enrichment. A Waters nano-Acquity M-Class dual pumping UPLC system (Milford, MA) was configured for on-line trapping of a 5 µL injection at 3 µL/min with reverse-flow elution onto the analytical column at 300 nL/min. Columns were packed in-house using 360 µm o.d. fused silica (Polymicro Technologies Inc., Phoenix, AZ) with 5-mm sol-gel frits for media retention and contained Jupiter C18 media (Phenomenex, Torrence, CA) in 5 µm particle size for the trapping column (150 µm i.d. x 4 cm long) and 3 µm particle size for the analytical column (75 µm i.d. x 70 cm long). Mobile phases consisted of (A) 0.1% formic acid in water and (B) 0.1% formic acid in acetonitrile with the following gradient profile (min, %B): 0, 1; 2, 8; 20, 12; 75, 30; 97, 45; 100, 95; 110, 95; 115, 1; 150, 1.

***Protein extraction:*** Filters were cut using sterile, disposable scalpels in a weigh boat that was on wet ice. The filters post-cell enrichment were transferred to a 15-mL centrifuge tube safe for chloroform:methanol extraction (Genessee Scientific, El Cajon, CA) and the tube placed in ice. The original cryovial tube was rinsed with 2 mL of 50:50 methanol:water (MeOH:H_2_O), vortexed and the rinse transferred to the respective 15-mL tube containing cut up filter pieces. The samples were then rotated end-over-end for 5 minutes at RT and placed back on ice. The following solvents were added, vortexing in between: 2.0-mL ice-cold H_2_O, 3.0-mL ice-cold MeOH and 8.0-mL of ice-cold chloroform (CHCl_3_). Following the CHCl_3_ addition, tubes were vortexed for 1 min and placed on ice for 5 min. Tubes were centrifuged at 5,000 *g* for 10 min at 4⁰C. The interphase layer was transferred into a chloroform-safe 1.7-mL microcentrifuge tube (Sorenson Safe Seal, Salt Lake City, UT). 1.0-mL of ice-cold MeOH was added to each sample, the samples were vortexed for ~10 sec, and then centrifuged at 18,000 *g* at 4⁰C for 10 min. Without disturbing the white pellet on the bottom of each tube, the MeOH was poured off, and the wash step with 1-mL of MeOH, vortexing and centrifugation was repeated. Following the second MeOH wash, the pellet was dried briefly in a SpeedVac vacuum concentrator (ThermoFisher Scientific, Waltham, MA). Each pellet was then resuspended in 100 µL of 8M urea in 100 mM NH_4_HCO_3_ and the protein quantity was measured using a Bicinchoninic Acid (BCA) protein assay (Thermo Fisher Pierce, Rockford, IL). An appropriate volume of 500 mM dithiothreitol (DTT, Thermo Fisher Pierce) was added to obtain a 10 mM concentration in the sample. The samples were incubated at 37⁰C for 1 h shaking (800 rpm) on a Thermomixer (Eppendorf, Happauge, NY). The samples were then diluted 8-fold with 100 mM NH_4_HCO_3_ and sufficient 1 M CaCl_2_ was added to obtain 1 mM concentration in each sample. 1 µg of trypsin was added to each sample and incubated for 3 h at 37⁰C shaking (800 rpm) on a Thermomixer. C18 solid phase extraction (SPE) was performed on each sample using 1 mL/50 mg columns (Strata C-18E, Phenomenex, Torrance, CA). The columns were pre-blocked using 100 µg of bovine serum albumin before the samples were applied. The eluted peptides were concentrated, a BCA protein assay was performed, and the samples were diluted to 0.1 µg/µL for MS analysis.

***Mass spectrometry:*** Mass-spectrometry (MS) analysis was performed using a Q-Exactive Plus mass spectrometer (Thermo Scientific, San Jose, CA) outfitted with a home-made nano-electrospray ionization interface. Electrospray emitters were prepared using 150 µm o.d. x 20 um i.d. chemically etched fused silica. The ion transfer tube temperature and spray voltage were 300ºC and 2.2 kV, respectively. Data were collected for 100 min following a 15 min delay from sample injection. FT-MS spectra were acquired from 300-1800 m/z at a resolution of 35k (AGC target 3x106) and the top 12 FT-HCD-MS/MS spectra were acquired in data dependent mode with an isolation window of 2.0 m/z and at a resolution of 17.5k (AGC target 1x105) using a normalized collision energy of 30 and a 30 sec exclusion time.

MS-GF+ version v2017.01.13 was used to identify peptides from the LC-MS/MS spectra. Search parameters included a +/-20 ppm parent mass tolerance, partial trypsin rules, and dynamic oxidized methionine residues. The candidate protein list was assembled by combining the *Pseudoalteromonas* sp. 13-15 proteome (3589 proteins) with the 6-frame translation of phage PSA-HP1 (161 proteins), the 6-frame translation of phage PSA-HS2 (125 proteins), and a collection of 195 contaminant proteins. The spectra were filtered with an MSGF E-value score of <1x10^-9^ and 2 or more unique peptide sequences were required to consider a protein identified. Passing spectra were counted and used as a relative abundance value for comparing across datasets.

Proteomics data are available at MassIVE and the ProteomeXchange repositories with accession numbers MSV000083626 and PXD013204, respectively.

***Quality assessment:*** A quality analysis was performed (**Supplementary Fig. S10-S12**) as previously described (1). The proteome samples that didn’t meet the expected thresholds of at least three of these metrics were excluded from downstream analyses (**Supplementary Fig. S10-S12**). In total, these low-P samples were excluded: “Con_T060_M_R2”, “Con_T100_M_R1”, “HP1_T080_M_R1”, and “HS2_T060_M_R3”. The first metric is the richness (number of distinct proteins) per sample, with the expectation that the within-replicates richness doesn’t deviate more than ±10% of the average richness of the three replicates. Richness was determined using the “specnumber” function of the package “vegan” in R. The second metric is the distance between samples in a Principal Coordinate analysis conducted on a Bray-Curtis dissimilarity matrix of the proteomes, with the expectation that all samples would fall within the 95% confidence interval for the same growth conditions upon challenging these proteomes with a different decoy proteome dataset generated from different growth conditions. The Bray-Curtis dissimilarity matrix was calculated using the function “vegdist” with the method “bray”, and the ordination was conducted using the function “capscale” with no constraints. The 95% confidence intervals were determined using the standard deviation method in the function “ordiellipse”; all of the three functions are part of the package “vegan” in R. The third metric is the module membership after hierarchical clustering followed by Dynamic Tree cutting (a top-down algorithm) or Dynamic Hybrid tree cutting (a bottom-up algorithm) (7), with the expectation that no sample should cluster with the decoy proteome dataset or emerge as a sole member of a module (singleton). Hierarchical clustering was conducted using the “pvclust” function with 10,000 bootstrap iterations and using the average as the clustering method and the correlations for calculating the distances. Tree cutting was conducted using the “cutreeDynamic” function with a minimum cluster size of 3 and using the “tree” and “hybrid” methods; both functions are part of the package “pvclust” in R. The last metric is the pairwise correlations between the replicates of the same treatment and time point, with the expectation that the correlations should be as close as possible to 1. The correlations were calculated using the Pearson’s correlation method and correlations that were <0.97 were considered for exclusion after consulting the results from the other metrics.

## Lipidomics:

For the extractions of intracellular lipids and metabolites, MPLEx extraction was used for the analysis of the intracellular metabolites and lipids described herein. This is a modified bi-phasic solvent extraction protocol previously described and used (8,9). Frozen samples on the membrane were transferred to microcentrifuge tubes and the metabolites and lipids were extracted with a mixture of chilled (-20°C) chloroform/methanol (2:1, v/v) also with nanopure water. Then, the samples were repeatedly vortexed to ensure thorough mixing. The samples were centrifuged at 15,000 *g* for 10 min at 4°C to separate aqueous and organic layers from denatured protein pellets. Aliquots of the upper aqueous layer containing polar metabolites and bottom organic layer with the total lipid extract (TLE) were transferred to glass vials and completely dried. The polar metabolite dried extracts were stored at -20°C until GC-MS. The TLE was reconstituted in 2:1 chloroform/methanol and stored at -20C until LC-MS analyses.

For processing, the TLEs were analyzed as outlined in (10). A Waters Acquity UPLC H class system interfaced with a Velos-ETD Orbitrap mass spectrometer was used for LC-ESI-MS/MS analyses. The TLEs were dried and then reconstituted in 40 μl of methanol of which 10 μl was injected onto a Waters CSH column (3.0 mm x 150 mm x 1.7 μm particle size) and separated over a 34-minute gradient (mobile phase A: ACN/H2O (40:60) containing 10 mM ammonium acetate; mobile phase B: ACN/IPA (10:90) containing 10 mM ammonium acetate) at a flow rate of 250 μl/min. Eluting lipids were introduced to the MS via electrospray ionization only in negative mode given that the subclasses present in the samples all ionize in this mode. Lipids were fragmented using higher-energy collision dissociation (HCD) and collision-induced dissociation (CID).

Lipid identifications were made using LIQUID (10). Confident identifications were selected by manually evaluating the MS/MS spectra for diagnostic and corresponding acyl chain fragments of the identified lipid. In addition, the precursor isotopic profile, extracted ion chromatogram, and mass measurement error along with the elution time were evaluated. To facilitate quantification of lipids, a reference database for lipids identified with LIQUID was created and features from each analysis were then aligned to this reference database based on their identification, m/z and retention time using MZmine 2 (11). Aligned features were manually verified and peak apex intensity values were exported for subsequent statistical analysis.

## Endometabolomics:

The extraction was the same as for the lipid samples described above. Then, analyses were done as reported previously (12). Briefly, extracted metabolites were completely dried with Speed-Vac concentrator (CentriVap, LABCONCO, Kansas City, MO) and chemically derivatized for GC-MS (Agilent 7890A gas chromatograph coupled with a single quadrupole 5975C mass spectrometer, Agilent Technologies, Inc, Santa Clara, CA). All the sample vials were randomized in the running order during the chemical derivatization and instrumental analysis, and the samples were analyzed by the instrument within 24 hours after the derivatization. All the collected mass spec data files were converted to netCDF format and processed using Metabolite Detector (13). All the peaks were matched to PNNL augmented version of Agilent metabolomics database which has retention index and fragmented spectra of metabolites, and additionally cross-checked with Wiley Registry 11th Edition and NIST17 GC-MS spectral databases. Identification of detected metabolites were validated manually to avoid misidentification of metabolites or false positive and negative errors. Peak intensity from the blanks was subtracted from the samples. Peak area values of detected metabolites were log-transformed for further statistical analysis.

## RNA-to-protein ratio calculations:

# The RNA:protein was calculated by dividing the average normalized transcript abundance (FPKM) by the average normalized protein abundance, both averaged across three replicates. There was no significant difference between protein extracted from each treatment.

**DOC chemical composition in the exometabolome:**

To remove salts commonly found in culturing media that can interfere with electrospray ionization (ESI) for high resolution mass spectrometry, solid phase extraction (SPE) was performed on the filtered exometabolome/DOC aqueous samples using a protocol adapted from (14). Briefly, 3 mL Bond Elut PPE cartridges (Agilent) were prepped with 3 mL of laboratory grade MeOH. PPL cartridges (Agilent Bond Elut PPL) were used to capture analytes ranging in polarity (polar to non-polar) due to the cartridge composed of a styrene-divinylbenze polymer modified with a proprietary nonpolar surface. Samples were acidified to pH 2 using 1M HCl to enhance extraction efficiency. Analytes were then extracted by passing a total sample (~50 mL) through cartridges under vacuum, followed by 5x washes with 0.01 M HCl solution, for a total washing volume of 15 mL, to remove residual salts captured in cartridge sorbent. Cartridges were removed from vacuum and cartridge sorbents containing analytes were dried completely using filtered air. Finally, analytes were eluted off dried cartridge sorbent using 1.5 mL MeOH into 2 mL glass vials and stored in -80˚C until high resolution-mass spectrometry analysis.

***Direct Inject FTICR-MS*:** High resolution mass spectra of the SPE exometabolome filtrate were collected by direct injection using a Bruker 9.4-Tesla Fourier transform ion cyclotron resonance (FTICR) mass spectrometer located at University of Arizona. A standard Bruker electrospray ionization (ESI) source was used to generate negatively charged molecular ions. Samples were then introduced directly to the ESI source. The instrument settings were optimized by tuning on a Suwannee River fulvic acid (SRFA) standard, purchased from International Humic Substances Society (IHCC). Blanks (HPLC grade methanol) were analyzed at the beginning and end of the day to monitor potential carry over from one sample to another. The instrument was flushed between samples using a mixture of water and methanol. The ion accumulation time (IAT) was varied to account for differences in C concentration between samples. One hundred and forty‐four individual scans were averaged for each sample and internally calibrated using an organic matter homologous series separated by 14 Da (CH_2_ groups). The mass measurement accuracy was <1 ppm for singly charged ions across a broad *m/z* range (100–1,200 m*/z*). The mass resolution was ~240 K at 341 m*/z*. The transient was 0.8 s. Data Analysis software (BrukerDaltonik version 4.2) was used to convert raw spectra to a list of *m/z* values applying FTICR‐MS peak picker module with a signal‐to‐noise ratio (S/N) threshold set to 7 and absolute intensity threshold to the default value of 100. After assigning peaks (see next paragraph), a cluster analysis with the blanks and the rest of the samples was performed using MetaboAnalyst 5.0 (15), which involves normalizing the data by the median, applying a log-transformation, and scaling by Pareto scaling, with a Eucledian-based clustering distance and choosing the ‘ward’ clustering method. The media-only blank clustered in a separate group from the rest (**Supplementary Figure S13**) and, as FTICR data is based on presence or absence of compounds, downstream analyses were continued with the samples alone.

***Compound identification*:** Putative chemical formulas were assigned using Formularity software (16). Chemical formulas were assigned based on the following criteria: S/N > 7, and mass measurement error < 1 ppm, taking into consideration the presence of C, H, O, N, S and P and excluding other elements. Peaks with large mass ratios (*m*/*z* values > 500 Da) often have multiple possible candidate formulas. These peaks were assigned formulas through propagation of CH_2_, O, and H_2_ homologous series. Additionally, to ensure consistent choice of molecular formula when multiple formula candidates are found the following rules were implemented: the formula with the lowest error with the lowest number of heteroatoms was consistently picked and the assignment of one phosphorus atom required the presence of at least four oxygen atoms. Biochemical compound classes were reported as relative abundance values based on counts of C, H, and O for the following H:C and O:C ranges: lipid-like (0 < O:C ≤ 0.3 and 1.5 ≤ H:C ≤ 2.5), unsaturated hydrocarbon-like (0 ≤ O:C ≤ 0.125 and 0.8 ≤ H:C < 2.5), peptide-like (0.3 < O:C ≤ 0.55 and 1.5 ≤ H:C ≤ 2.3), amino sugar-like (0.55 < O:C ≤ 0.7 and 1.5 ≤ H:C ≤ 2.2), polyphenol-like (0.125 < O:C ≤ 0.65 and 0.8 ≤ H:C < 1.5), carboxylated/oxygen rich (0.65 < O:C ≤ 1.1 and 0.8 ≤ H:C < 1.5), and condensed hydrocarbon-like (aromatics; 0 ≤ 200 O:C ≤ 0.95 and 0.2 ≤ H:C < 0.8) (17). After all *m/z* were assigned putative chemical classes based on OtoC and HtoC ratios each chemical class was normalized based on total peaks within each sample. This allows for more confident statistical analysis on normalized relative abundances of chemical compound classes per sample. Boxplots and Wilcoxon non-parametric rank sum statistical tests were performed within lipids and peptides’ relative abundances using R (version 3.6.1).

***Gibbs Free Energy***: Gibbs free energy (GFE) was calculated, as described previously (18) including only peaks that were assigned a putative molecular formula in all samples. Briefly, each assigned molecular formula was used to calculate the nominal oxidation state of C (NOSC) according to the following equation:

1. NOSC = - ((4xC+H-3xN-2xO+5xP-2xS)/C) + 4

NOSC values were then used to calculate the GFE of each compound according to equation (2). Low GFE values are an indication of compounds more thermodynamically favored to undergo metabolic and chemical reactions (i.e. available for microbial communities), whereas higher values reflect microbially degraded and transformed (i.e. recalcitrant) OM. Average GFE boxplots and density distribution based on counts between high-P and low-P samples were then plotted using R (version 3.6.1). Wilcoxon rank sum tests (non-parametric) were performed between high-P and low-P GFE values to determine the significance of the observed differences between GFE of each group.

1. DeltaG Cox = 60.3 - 28.5 x (NOSC)

***Network Heterogeneity:*** Organic matter transformation analysis for each individual sample replicate was done via network analysis using MetaNetter (19), a Cytoscape Version 3.8.0 plug‐in, as described previously (20). Briefly, mass differences between the two *m/z* values resulting from a chemical transformation were demonstrated as an edge in the network, and the initial and final compounds (namely, reactant and product) as nodes. For example, the gain or loss of CO_2_ (i.e., mass difference Δ*m/z* = 43.9898) between two compounds would be represented as two ‘nodes’ connected by an edge named ‘CO_2_’. For each transformation, there may be a series of connected *m/z* values, and one *m/z* value may be connected to other *m/z* values by more than one transformation. For a biochemical reaction to be considered valid by this method, both the reactant and the product should be present. Network analyzer was then performed on all *m/z* transformations to calculate network heterogeneity of each sample. The network heterogeneity reflects the tendency of a network to contain hub nodes where a hub is a node with a number of transformations that greatly exceeds the average. Thus, higher network heterogeneity signifies a larger and more diverse rate of node connections, signifying a more transformed system (i.e. the same compound could be produced through a multitude of reactions and/or the same compound can be degraded in many different pathways). Statistical analysis (Wilcoxon rank sum test) was performed with all sample network heterogeneity values compared in each infection between media type and plotted using R (version 3.6.1). Relative overall change in network heterogeneity media differences between virocells was calculated by first taking the median (non-parametric dataset) of each virocell in high-P and low-P conditions. Median low-P values were subtracted from high-P values to get the overall difference in network heterogeneity between media in each virocell. Finally, the HP1-virocell difference was divided by the HS2-virocell difference to compare the overall change in media network heterogeneity values between virocells.

**Principal component analysis:**

PCA was performed on all omics data types independently, from normalized data averaged across biological replicates. Briefly, normalization for each data type is as follows: transcriptomes were normalized to FPKM as described above; proteomics data was calculated as z-scores across all samples, treatments, and infections; endometabolomic media blanks were subtracted from the sample data; both lipidomic and endometabolomic data were mean-centered by lipid or metabolite; exometabolomic intensities of each m/z normalized by sum within a single sample. Due to the nature of high zero-inflated data in environmental exometabolomics datasets, masses were analyzed that were 50% ubiquitous across the entire sample matrix in order to avoid overfitting of zeros common to PCA multivariate analysis. All PCAs were calculated using ‘prcomp’ in R.

**Statistics:**

Beyond the omics-specific methods and linear mixed effects models explained above, all mentions of significance in the exometabolome section come from Wilcoxon (or Mann-Whitney) tests unless otherwise mentioned. The mentions of significance for relative protein abundances come from 2-tailed t-tests. RNA-to-protein ratios are evaluated with Spearman correlations. The impact of media (high-P or low-P) and phage (HP1 or HS2) on each ‘omics data type was evaluated with a marginal ANOVA analysis.


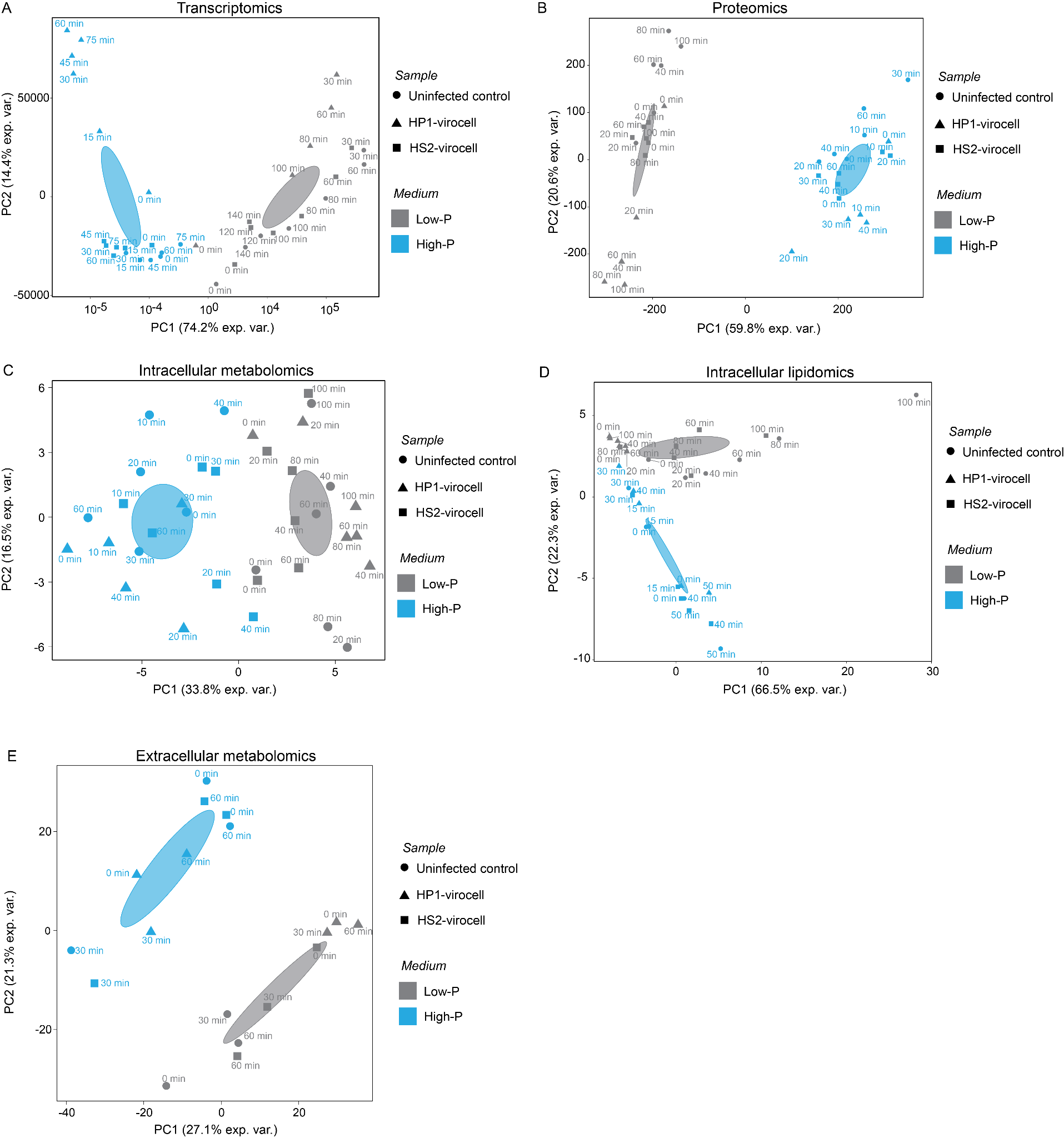


**Figure S1: Principal component analysis of each omics data type.** Ellipses represent 95% confidence levels based on high-P (blue) vs low-P (gray) conditions. Each point is the average of three biological replicates. Axes denote the percent variation explained (exp. var.) by each principal component.

##
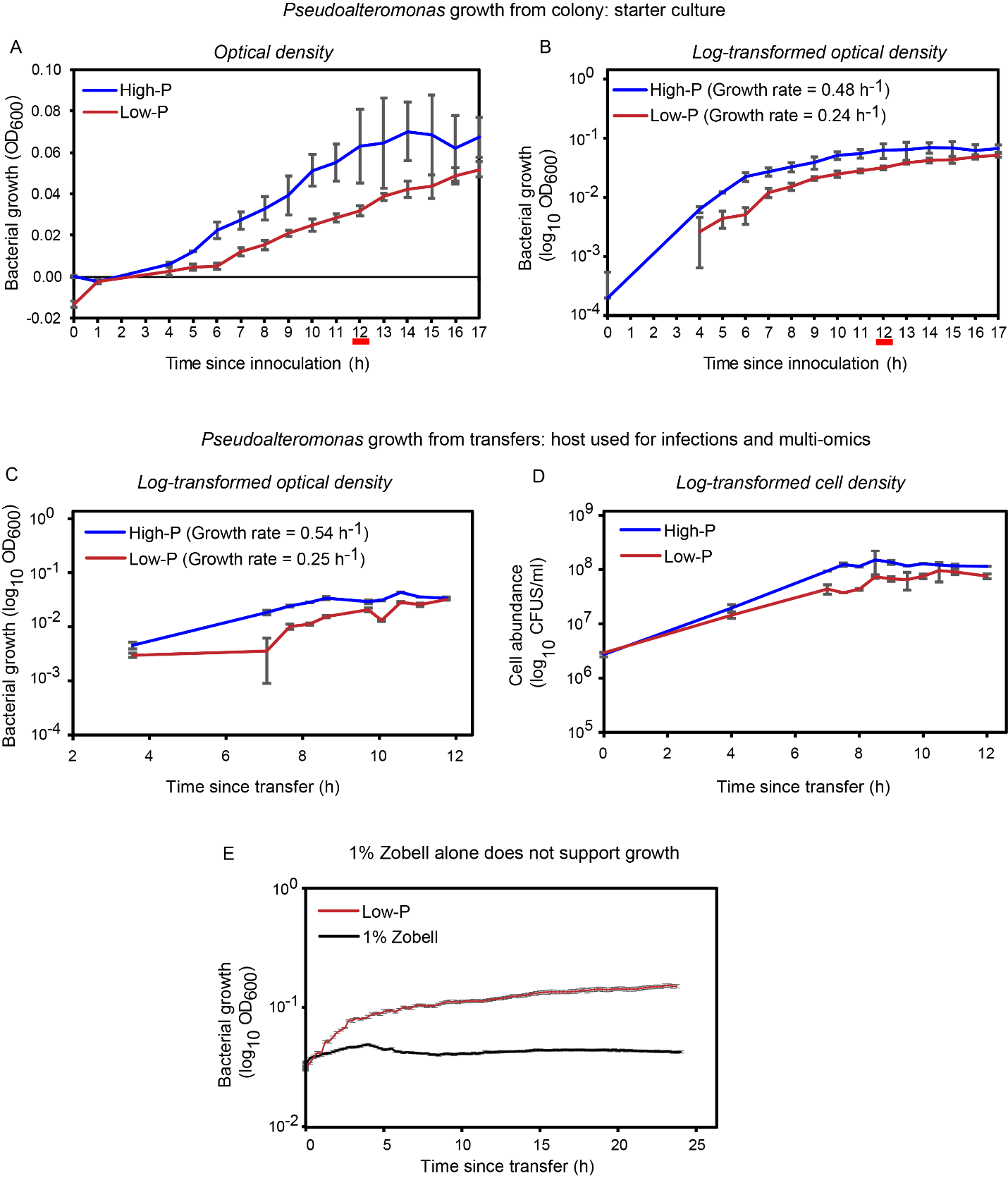


## Figure S2: Bacterial host growth in varying phosphate (P) conditions. *Pseudoalteromonas sp*. 13-15 growth from a colony into 10 mL of a high-P (1% Zobell + CNP) or a low-P (1% Zobell + CN) medium presented as raw optical density (OD) values (A) or log_10_-transformed (B). This represented the starter culture which, after 12 hours (as indicated by the red line), was transferred into 200 ml of the same medium (C, D) and enabled to grow until reaching ~10^8^ cells/ml to begin phage infections. Both log_10_-transformed OD values (C) and cell density (cfus/ml, D) were obtained for the transfer cultures. Growth rates are represented for both cultures (B, C). Cell growth was also tested in 1% Zobell with no added C, N, or P sources alongside the low-P medium (E), inoculated first from a colony as in A-B, then transferred as in C-D which is what is represented. For all plots, medium-only blank values were subtracted to the OD values of the growth treatments, and the average of 2-3 biological replicates and their standard error are plotted.


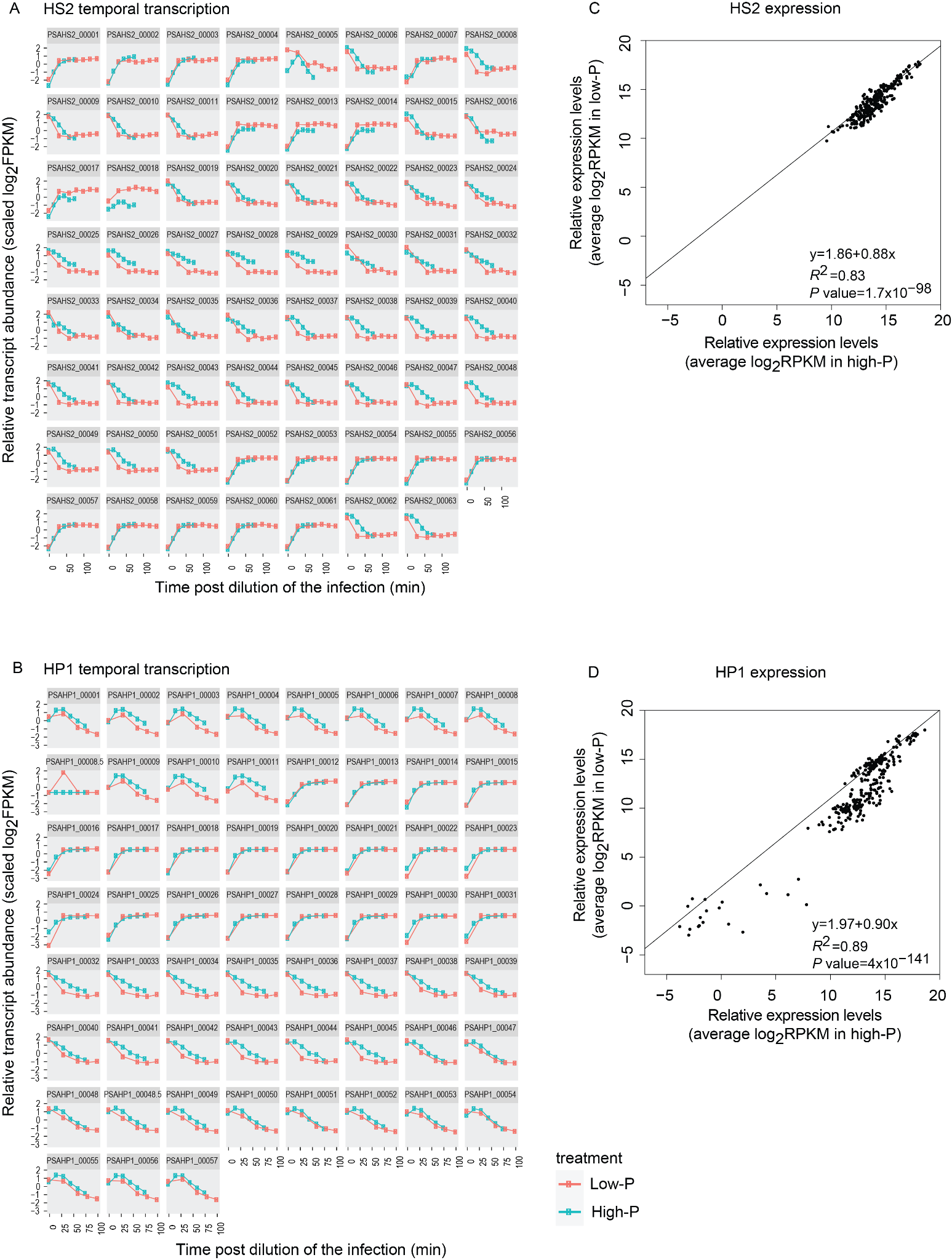


## Figure S3: Phage expression in high-P and low-P conditions. A) Temporal expression of phage HS2 genes overlapped in high-P and low-P conditions represented as the average of the log_2_FPKM for three replicates. B) Temporal expression of phage HP1 genes overlapped in high-P and low-P conditions represented as the average of the log_2_FPKM for three replicates. C) Pearson correlation between phage HS2 gene transcription values (normalized log_2_FPKM) in high-P versus low-P conditions. D) Pearson correlation between phage HP1 gene transcription values (normalized log_2_FPKM) in high-P versus low-P conditions.


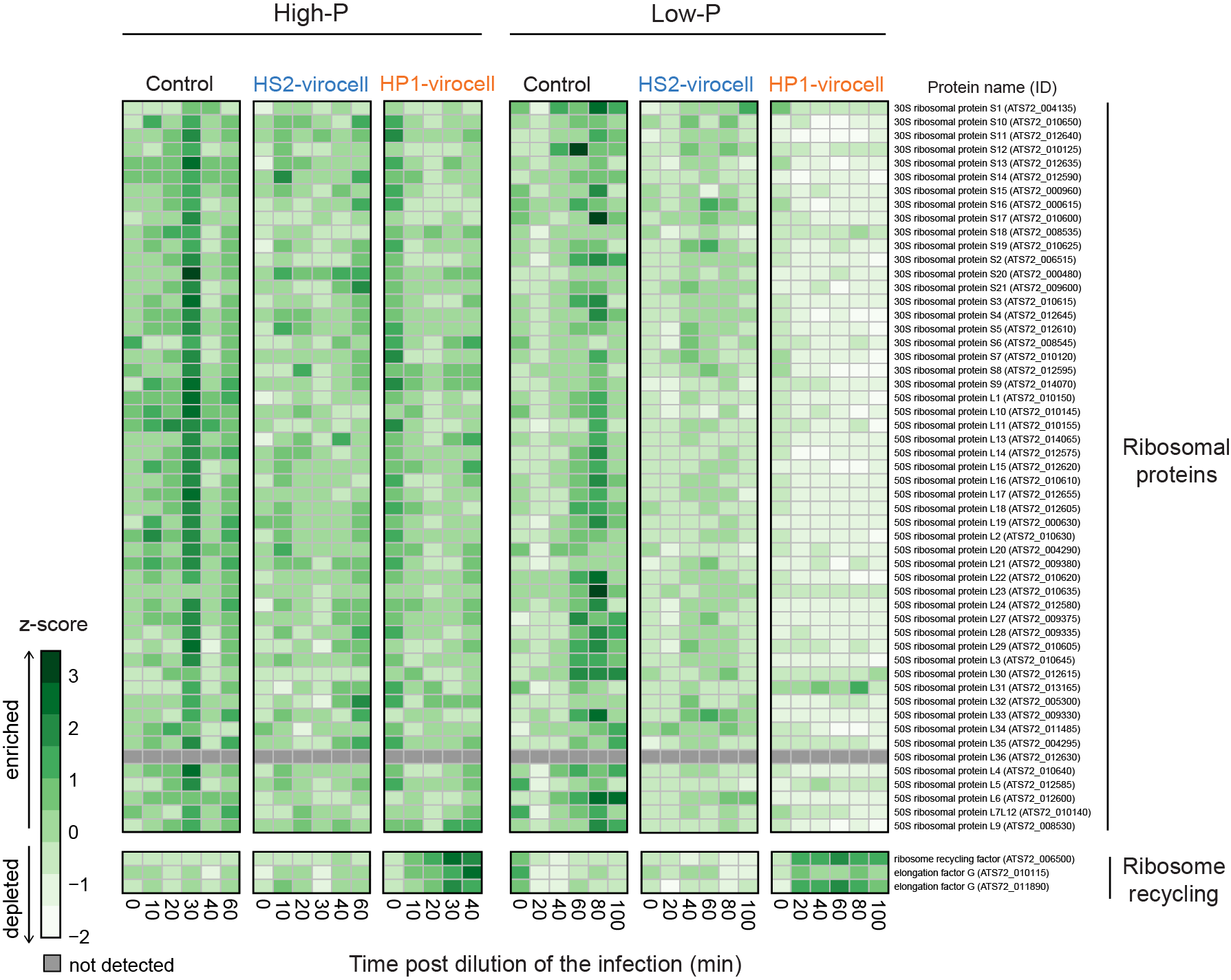


## Figure S4: Ribosomal proteins and ribosome recycling proteins in the virocells in high-P and low-P conditions. Heatmap of relative protein abundance of the ribosomal proteins and the ribosome recycling proteins in high-P and low-P conditions for uninfected control cells and both virocells, obtained from proteomics. Represented are z-scores. Proteins are considered enriched if z-score>0, depleted if z-score<0, and dark gray is a protein not detected in our dataset.

##
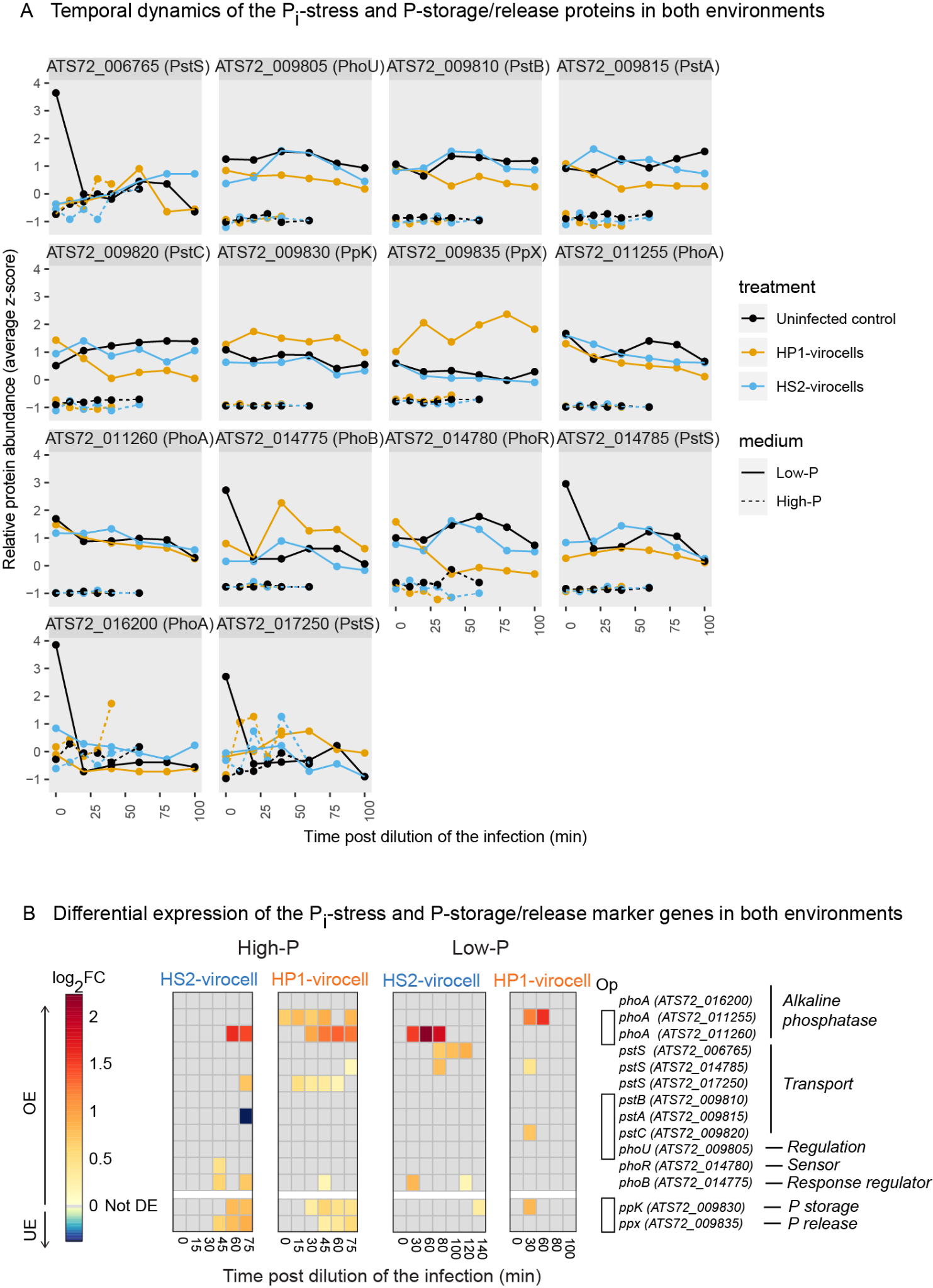


## Figure S5: Transcripts and proteins of P_i_-stress and P-storage/release genes in high-P and low-P conditions. A) Temporal dynamics of the P_i_-stress and the P-storage/release proteins, plotted as standardized abundances (average z-score for three biological replicates) in high-P (dashed lines) and low-P media (solid lines) for the virocells and uninfected control cells, obtained from proteomics. B) Heatmap with relative gene expression (log_2_FC relative to uninfected control cells in the same environment and time point) of the P_i_-stress and the P-storage/release genes in both media, obtained from transcriptomics. Genes are over-expressed (OE) if their log_2_FC>0, under-expressed (UE) if log_2_FC<0, and not DE if log_2_FC=0 (in gray). “Op” indicates if the genes are expected to be in an operon. Genes/proteins: phosphate ABC transporter 2C permease protein (PstA), phosphate ABC transporter ATP binding protein (PstB), phosphate ABC transporter permease (PstC), phosphate ABC transporter substrate binding protein (PstS), alkaline phosphatase (PhoA), phosphate regulon sensor histidine kinase (PhoR), DNA binding response regulator (PhoB), phosphate transport system regulator (PhoU), RNA degradosome polyphosphate kinase (PpK), and exopolyphosphatase (PpX).


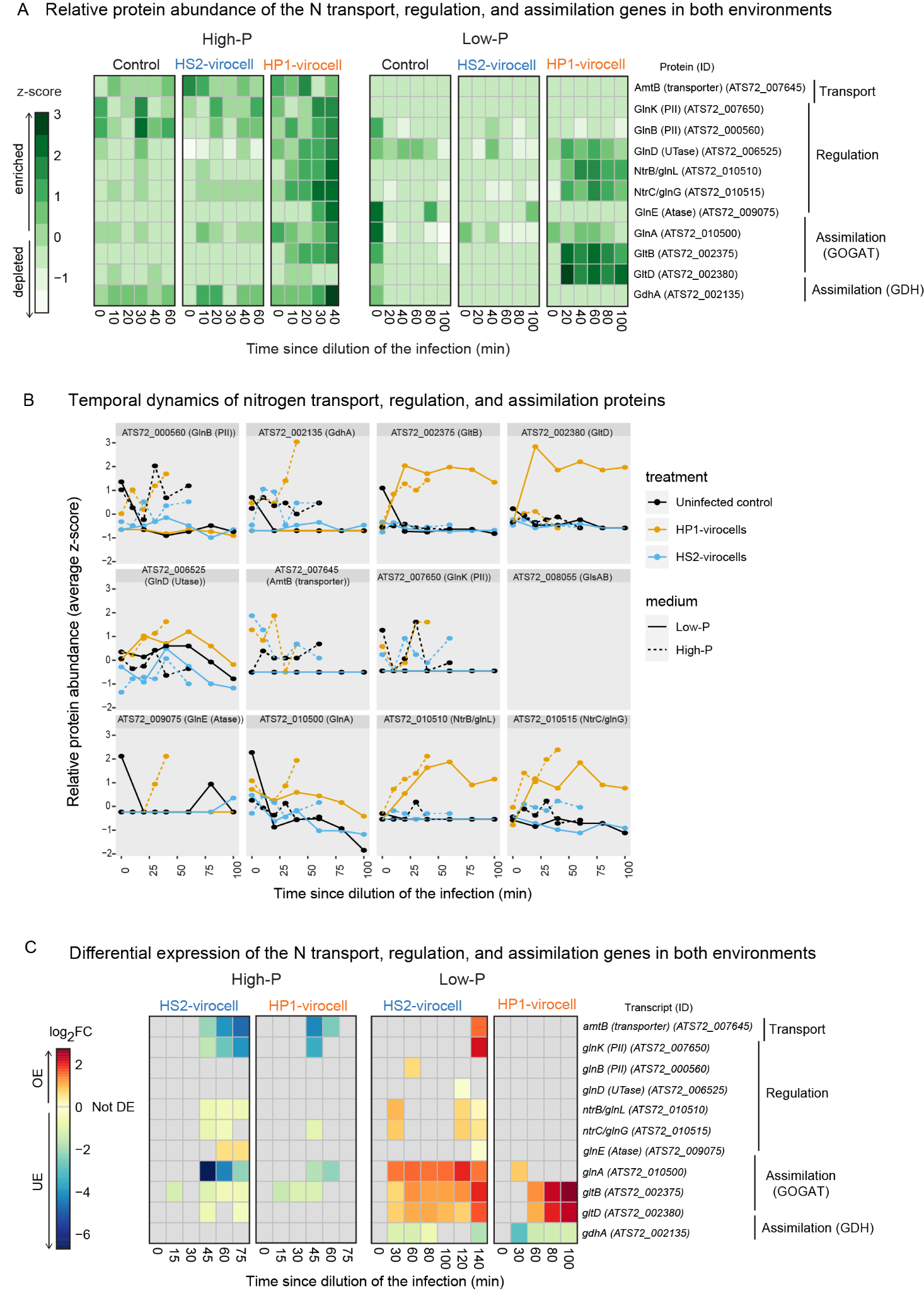


## Figure S6: Nitrogen transport, regulation, and assimilation transcripts and proteins in high-P and low-P conditions. A) Heatmap of relative protein abundance of the nitrogen (N) transport, regulation and assimilation genes in the high-P and low-P conditions for uninfected control cells and both virocells, obtained from proteomics. Represented are z-scores. Proteins are considered enriched if z-score>0 and depleted if z-score<0. B) Temporal dynamics of the N transport, regulation and assimilation proteins, plotted as standardized abundances (average z-score for three biological replicates) in high-P (dashed lines) and low-P (solid lines) media for the virocells and uninfected control cells, obtained from proteomics. C) Heatmap of expression of the N transport, regulation and assimilation genes in the high-P and low-P virocells relative to uninfected control cells in each respective condition, obtained from transcriptomics. Gray denotes genes that are not differentially expressed (not DE). “OE” is over-expressed, “UE” is under-expressed. *Genes/proteins*: transporter of ammonium (AmtB), nitrogen regulatory protein PII-2 (GlnK), Nitrogen Assimilation Two-Component System (NtrB and NtrC), nitrogen regulatory protein PII-1 (GlnB), protein-PII uridylyltransferase (GlnD), glutamine synthetase adenylyltransferase (GlnE), glutamine synthetase (GlnA), glutamate synthase (GltB and GltD), and glutamate dehydrogenase (GdhA). *Pathways*: glutamine oxoglutarate aminotransferase/glutamate synthase (GOGAT) and glutamate dehydrogenase (GDH).


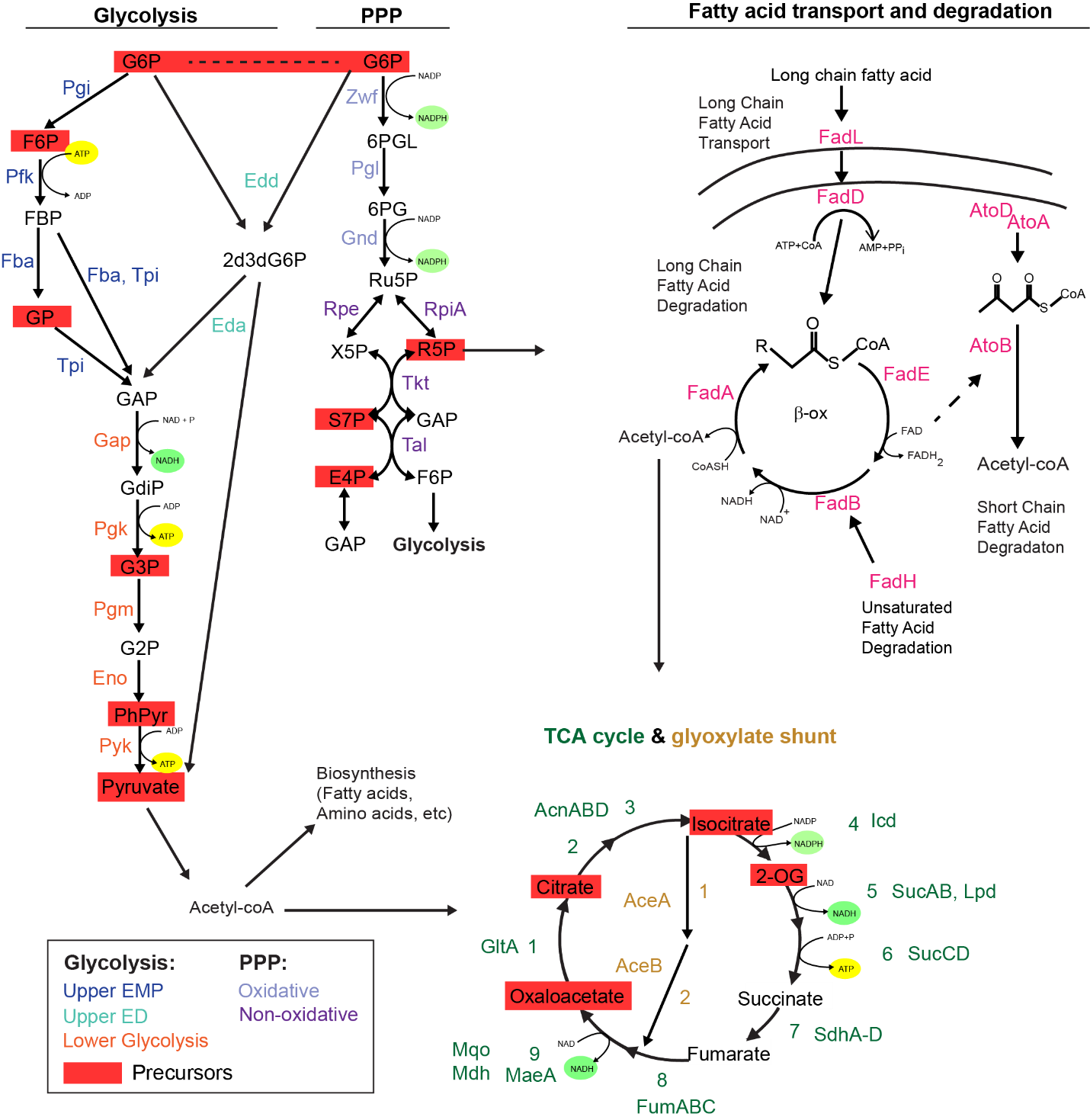


# **Figure S7: Schematic representation of some energy pathways in a bacterial cell.** Glycolysis, ED (Entner-Doudoroff), PPP (Pentose Phosphate Pathway), and TCA (Tricarboxylic acid) cycle from central carbon metabolism; and FA (Fatty acid) degradation. Glycolysis is most commonly known for the upper and lower EMP (Embden–Meyerhof–Parnas) pathway, but it also converges with ED, whereby upper ED is unique to ED, and lower ED is common to lower EMP, as defined by (21). The 13 precursor metabolites generated from central carbon metabolism are boxed in red. *Genes/proteins*: glucose-6-phosphate isomerase (Pgi), fructose-bisphosphate aldolase (FbaA), triose-phosphate isomerase (TpiA), glyceraldehyde-3-phosphate dehydrogenase (GapAB), phosphoglycerate kinase (PgK), phosphoglycerate mutase (GpmM), enolase (Eno), pyruvate kinase (PykA), phosphogluconate dehydratase (Edd), 2-keto-3-deoxygluconate-6-phosphate aldolase (Eda), glucose-6-phosphate dehydrogenase (Zwf), 6-phosphogluconolactonase (Pgl), 6-phosphogluconate dehydrogenase (Gnd), ribulose-phosphate 3-epimerase (Rpe), ribose-5-phosphate isomerase (RpiA), transketolase (TktAB), transaldolase (TalAB), dihydrolipoyl dehydrogenase (Lpd), citrate synthase (GltA), aconitate hydratase (AcnA), aconitate hydratase B (AcnB), 2 methylisocitrate dehydratase (AcnD), isocitrate dehydrogenase (Icd), 2-oxoglutarate dehydrogenase subunit E1 (SucA), dihydrolipoamide succinyltransferase (SucB), succinate CoA ligase subunit beta (SucC), succinate CoA ligase subunit alpha (SucD), succinate dehydrogenase flavoprotein subunit (SdhA), succinate dehydrogenase iron sulfur subunit (SdhB), succinate dehydrogenase 2C cytochrome b556 subunit (SdhC), succinate dehydrogenase 2C hydrophobic membrane anchor protein (SdhD), fumarate hydratase (FumA/FumB), class II fumarate hydratase (FumC), malate dehydrogenase (Mdh), malate:quinone oxidoreductase (Mqo I), isocitrate lyase (AceA), malate synthase (AceB), DNA-binding transcriptional dual regulator (FadR), long-chain-fatty-acid—CoA ligase (FadD), long-chain fatty acid outer membrane channel (FadL), 3-ketoacyl-CoA thiolase (FadA), enoyl-CoA hydratase (FadB), acyl-CoA dehydrogenase (FadE), 2,4-dienoyl-CoA reductase (FadH), 3-ketoacyl-CoA thiolase (FadI), 3-hydroxyacyl-CoA dehydrogenase (FadJ), beta complex (AtoA), acetyl-CoA acetyltransferase (AtoB), DNA-binding transcriptional activator (AtoC), and alpha complex (AtoD). *Metabolites*: glutamate (glu), glutamine (gln), glucose-6-phosphate (G6P), fructofuranose-6-phosphate (F6P), fructofuranose 1,6-bisphosphate (FBP), glycerone phosphate (GP), glyceraldehyde 3-phosphate (GAP), 3-phospho-glyceroyl phosphate (Gdip), 3-phospho-glycerate (G3P), 2-phospho-glycerate (G2P), phosphoenolpyruvate (PhPyr), 2-dehydro-3-deoxy-D-gluconate 6-phosphate (2d3dG6P), 6-phospho glucono-1,5-lactone (6PGL), gluconate 6-phosphate (6PG), ribulose 5-phosphate (Ru5P), xylulose 5-phosphate (X5P), ribose 5-phosphate (R5P), sedoheptulose 7-phosphate (S7P), and erythrose 4-phosphate (E4P).

#
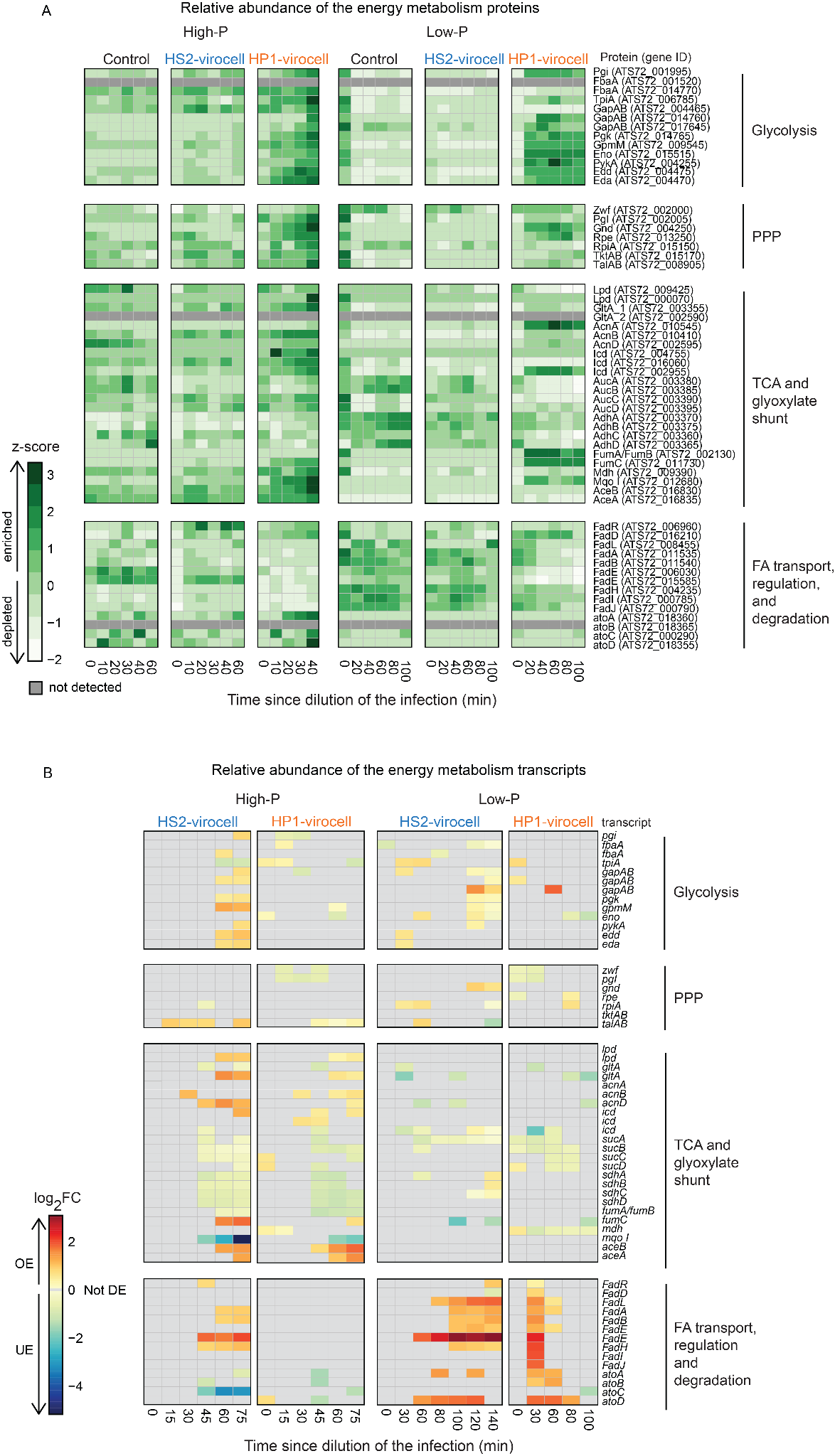


## Figure S8: Energy metabolism transcripts and proteins in high-P and low-P conditions. A) Heatmap of relative protein abundance in the high-P and low-P conditions for uninfected control cells and both virocells represented as z-score across all samples in both conditions, obtained from proteomics. Proteins are considered enriched if their z-score>0, and depleted if their z-score<0. Dark gray denotes proteins that are not detected in our dataset. B) Heatmap of expression of the genes calculated as log_2_FC in the virocells relative to uninfected control cells in the same condition and time point, obtained from transcriptomics. Genes are over-expressed (OE) if log_2_FC>0, under-expressed if log_2_FC<0, and not differentially expressed (not DE) if log_2_FC=0 (in gray). Gene, ID and functional assignment is the same for both heatmaps. Gene/protein names are in the legend of Figure S7.

**
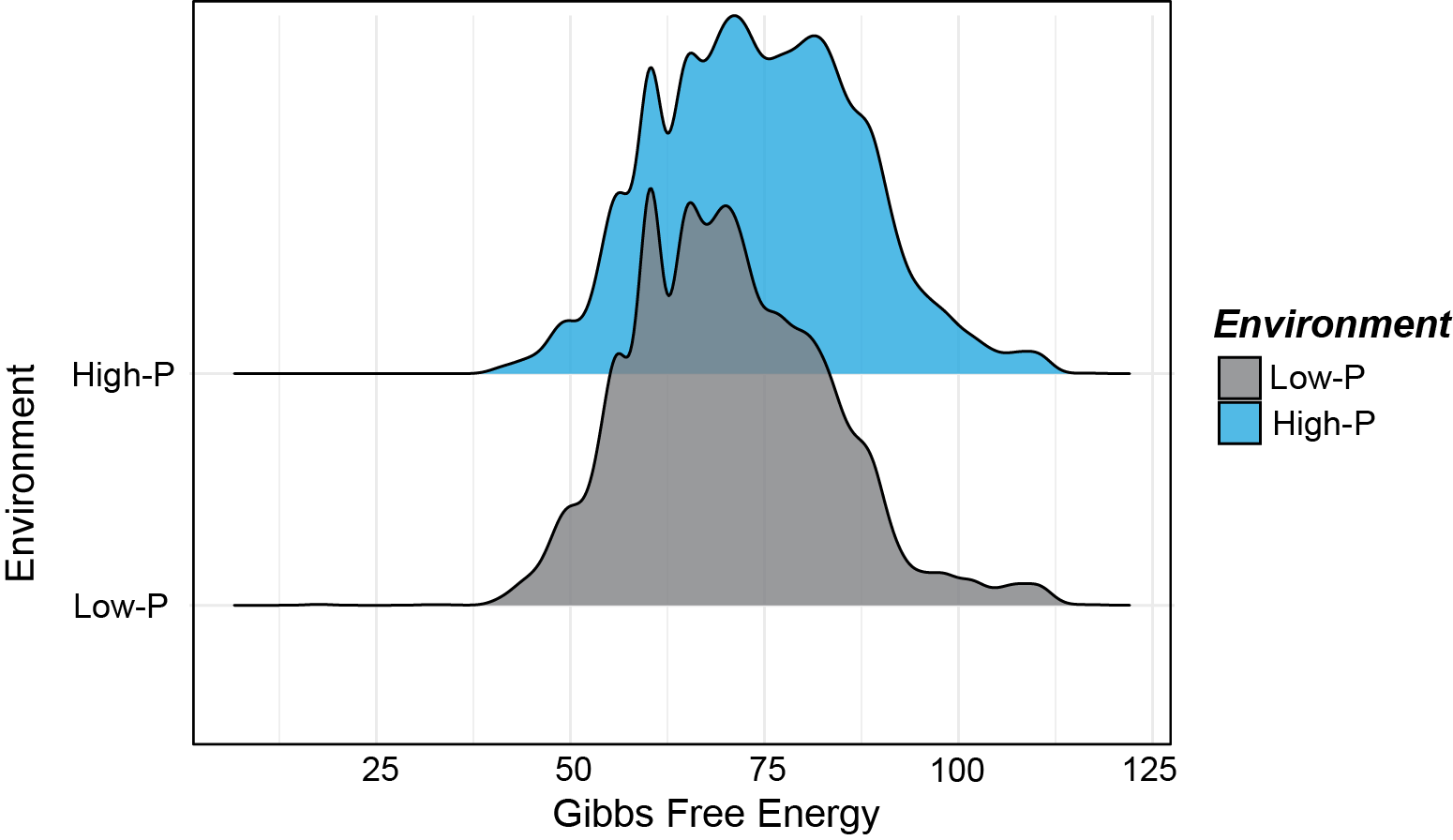
**

## Figure S9: Distribution of Gibbs Free Energy of all dissolved organic carbon chemical compounds between environments. Density plots of GFE distribution of all dissolved organic carbon (DOC) chemical compounds in the high-P (blue) and low-P (gray) conditions across all treatments and time points (0, 30, and 60 minutes post dilution of the infection). Each density plot is all the chemical compounds grouped and colored by environment. This plot leverages exometabolomic data.


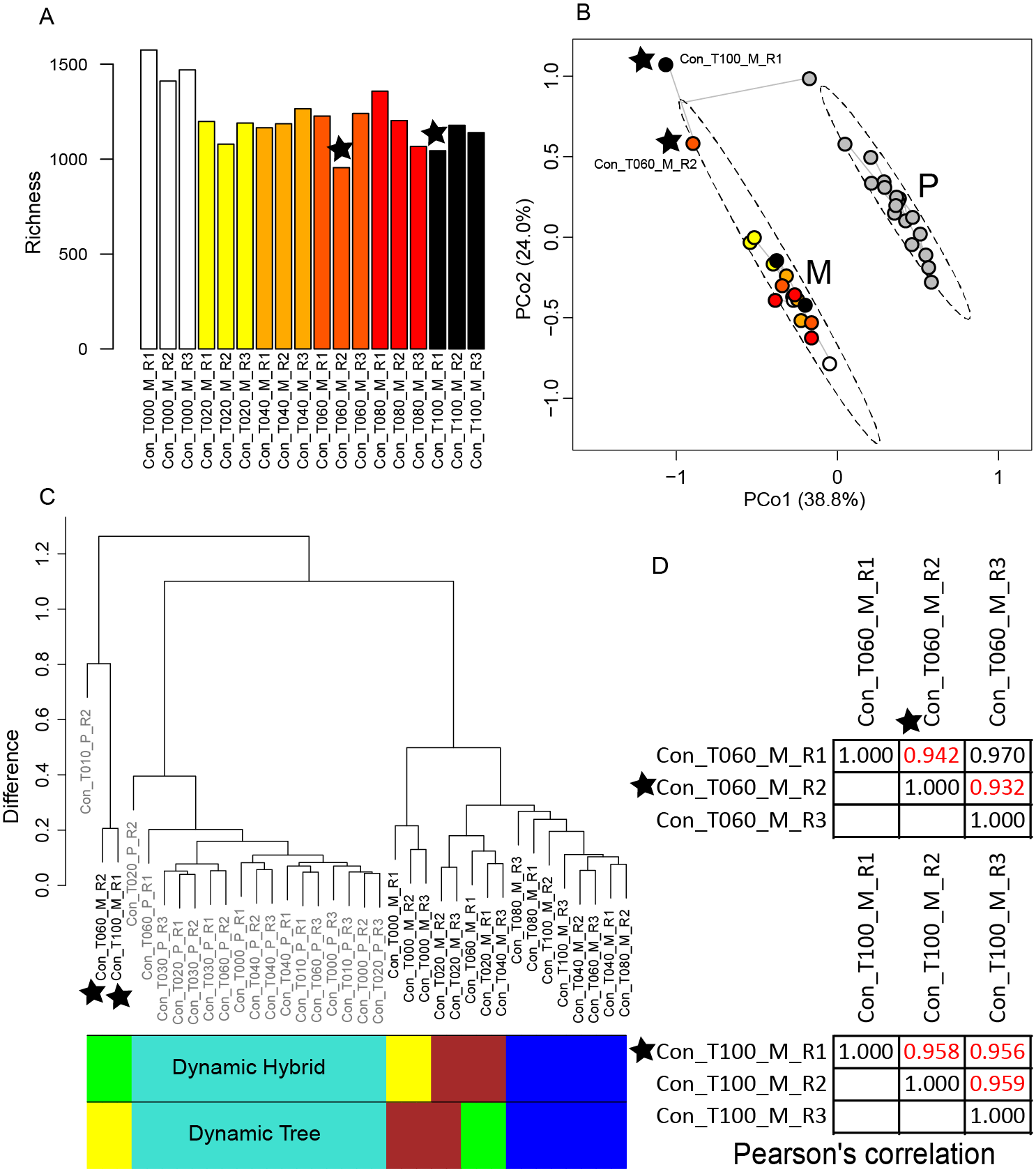


**Figure S10: Low-P proteome quality assessment in the uninfected control samples. A)** Barplot of the protein richness (number of distinct proteins) in each sample showing which samples have fewer total proteins relative to the other replicates at the same time point. A clear example of lower richness is the uninfected control sample taken 60 minutes after diluting the infection (T060) from the second biological replicate (R2). **B)** Principal Coordinate Analysis on a Bray-Curtis dissimilarity matrix of the proteomes showing that only a few samples fall outside the 95% confidence intervals (dashed ellipses) drawn around the centroid of each group. The “M” group is the low-P group of proteomes used in this study colored by their sampling time as indicated in panel (A). The “P” group is the high-P proteome dataset (1) used as a decoy for testing the within-group similarity of this study’s proteomes. The Con_T060_M_R2 and the Con_T100_M_R1 samples are the most distinct as they fall outside the 95% confidence intervals. **C)** Hierarchical clustering on a Bray-Curtis dissimilarity matrix of the proteomes using both the Dynamic tree cutting and the Dynamic Hybrid tree cutting methods showing that the Con_T060_M_R2 and the Con_T100_M_R1 samples clustered outside of the “M” group of proteomes by both methods. The colors represent the different clustering groups assigned by each method. **D)** Pairwise Pearson correlations of the replicates of the control proteome samples that were excluded from downstream analyses (“Con_T060_M_R2” and “Con_T100_M_R1”, highlighted by stars in panels (A-D)). Red text indicates a correlation that is less than 0.97.


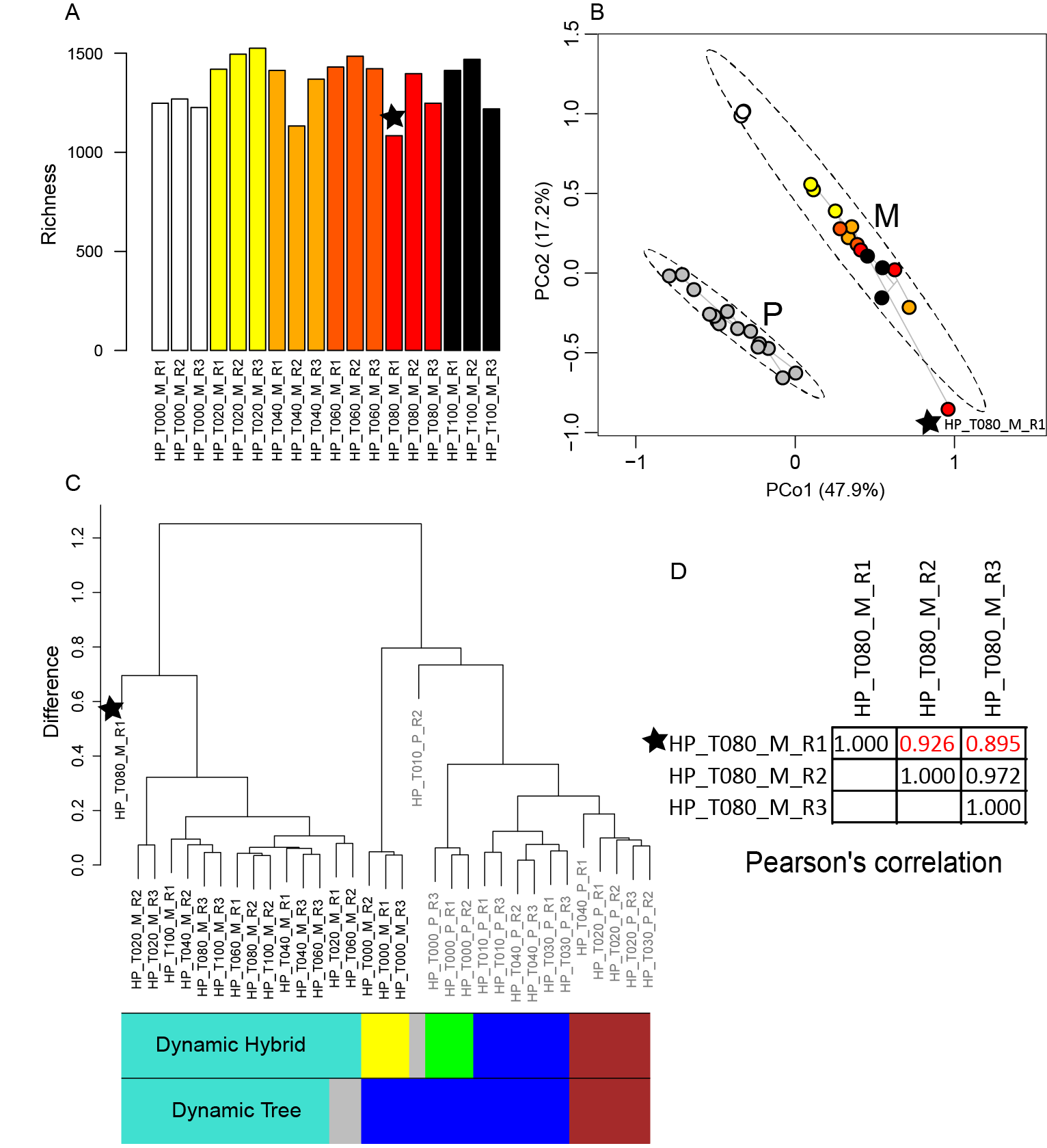


**Figure S11: Low-P proteome quality assessment in the HP1-virocell samples. A)** Barplot of the protein richness (number of distinct proteins) in each sample showing which samples have fewer total proteins relative to the other replicates at the same time point. A clear example of lower richness is the HP1-virocell sample (HP) taken 80 minutes after diluting the infection (T080) from the first biological replicate (R1). **B)** Principal Coordinate Analysis on a Bray-Curtis dissimilarity matrix of the proteomes with the 95% confidence intervals (dashed ellipses) drawn around the centroid of each group. The “M” group is the low-P proteome used in this study colored by their sampling time as indicated in panel (A). The “P” group is the high-P proteome dataset (1) used as a decoy for testing the within-group similarity of this study’s proteomes. The HP_T080_M_R1 sample is the most distinct as it falls outside the 95% confidence intervals. **C)** Hierarchical clustering on a Bray-Curtis dissimilarity matrix of the proteomes using both the Dynamic tree cutting method and the Dynamic Hybrid tree cutting methods. The colors represent the different clustering groups assigned by each method. **D)** Pairwise Pearson correlations of the replicates of the only HP1-virocell proteome sample that was excluded from downstream analyses (“HP_T080_M_R1”, highlighted by a star in panels (A-D)). Red text indicates a correlation that is less than 0.97.


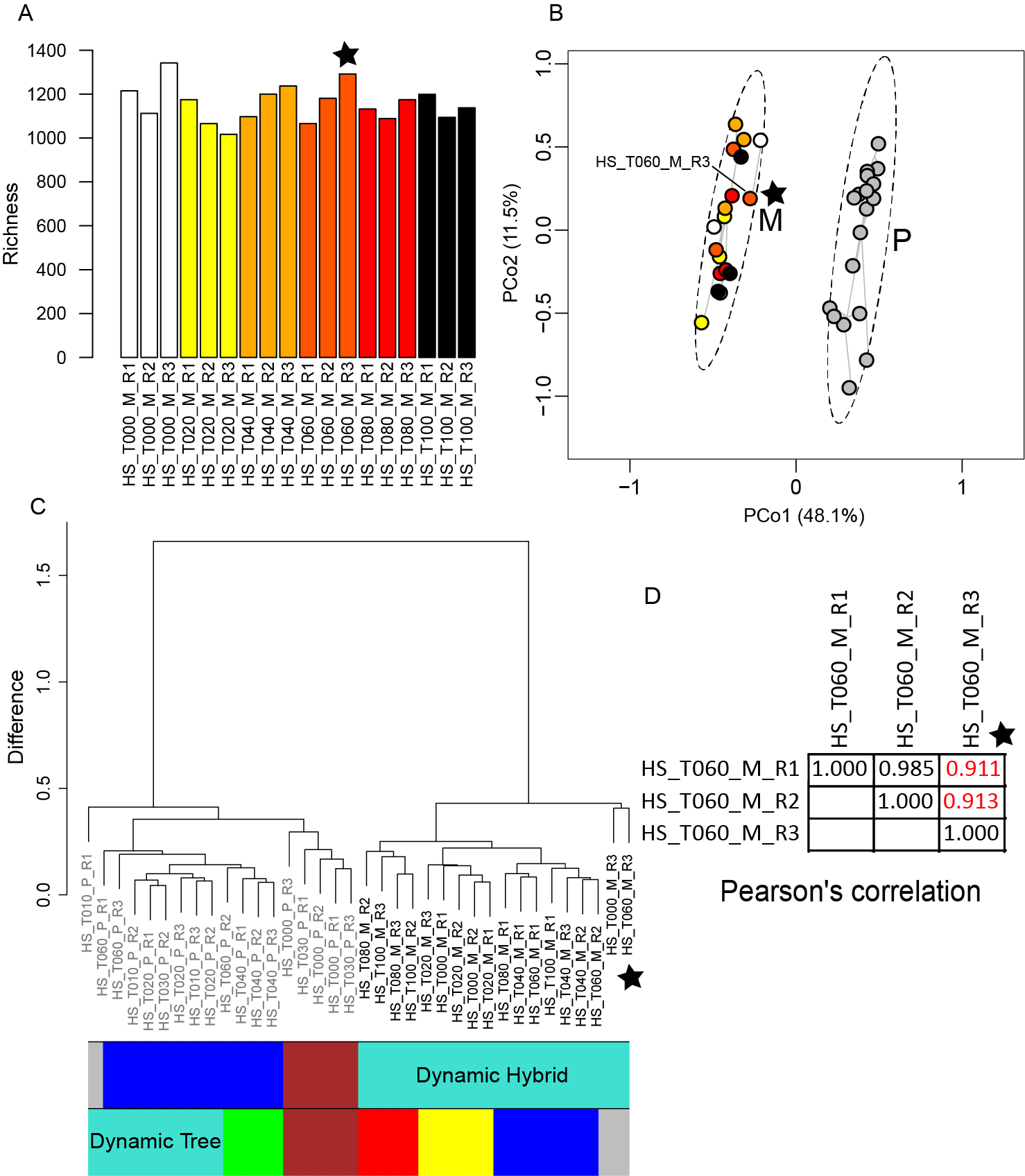


**Figure S12: Low-P proteome quality assessment in the HS2-virocell samples. A)** Barplot of the protein richness (number of distinct proteins) in each sample showing which samples have higher total proteins relative to the other replicates at the same time point. A clear example of higher richness is the HS2-infected sample (“HS”) taken 60 minutes after diluting the infection (T060) from the third biological replicate (R3). **B)** Principal Coordinate Analysis on a Bray-Curtis dissimilarity matrix of the proteomes with the 95% confidence intervals (dashed ellipses) drawn around the centroid of each group. The “M” group is the low-P proteome used in this study colored by their sampling time as indicated in panel (A). The “P” group is the high-P proteome dataset (1) used as a decoy for testing the within-group similarity of this study’s proteomes. The HS_T060_M_R3 sample falls close to the dashed line representing the 95% confidence intervals. **C)** Hierarchical clustering on a Bray-Curtis dissimilarity matrix of the proteomes using both the Dynamic tree cutting and the Dynamic Hybrid tree cutting methods. The colors represent the different clustering groups assigned by each method. The Dynamic tree cutting method shows that the HS_T060_M_R3 sample does not cluster with the rest of its group of samples. **D)** Pairwise Pearson correlations of the replicates of the only HS2-virocell proteome sample (HS_T060_M_R3) that was excluded from downstream analyses (highlighted by a star in panels (A-D)). Red text indicates a correlation that is less than 0.97.


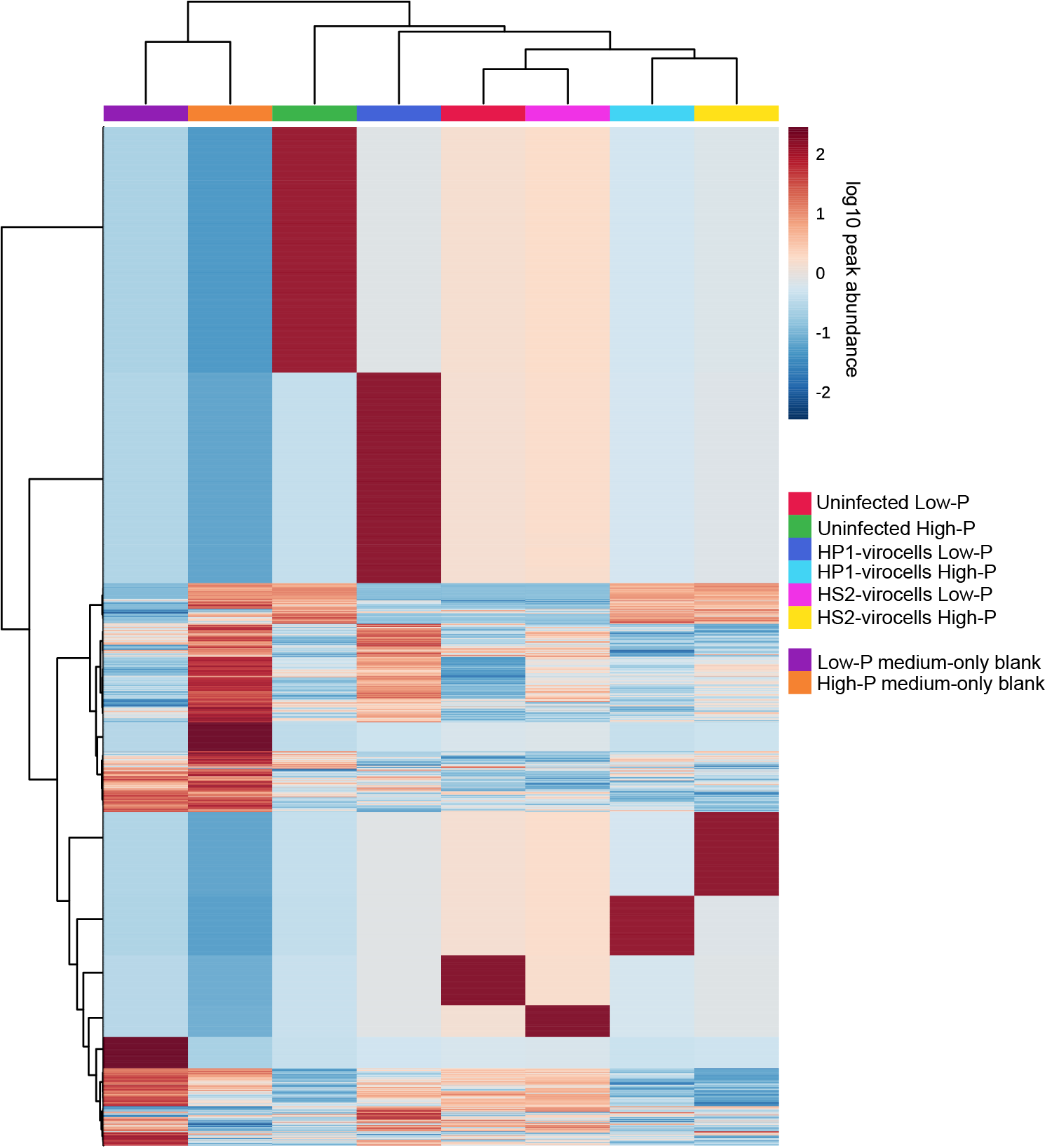


**Figure S13:** **Exometabolome sample clustering.** Separation between blanks and the rest of the exometabolome samples used in the study. Exometabolome samples were collected from uninfected cells in both media, infected cells in both media and medium-only blanks for each medium. Cluster analysis was conducted using MetaboAnalyst 5.0 (15), which normalizes the data by media, applied log_10_-transformation, and uses Euclidean distances and the Ward method for clustering.

**Table S1: Media composition.** Concentration of each nutrient tested in the high-P and low-P media used for the experiments, and of the phosphate species of two additional media batches from different ingredient lots.

|  | **μM TOP (total organic phosphate)** | **μM PO_4_ (inorganic phosphate)** | **μM NO_3_ (nitrate)** | **μM NH_4_ (ammonium)** | **μM SO_4_ (sulfate)** |
| --- | --- | --- | --- | --- | --- |
| Low-P medium (batch 1; used here) | 5.3 | 0.6 | 137.8 | 366655.6 | 28224 |
| High-P medium (batch 1; used here) | 55.1 | 47.8 | 203.6 | 275669.4 | 21399.1 |
| Low-P (batch 2) | 7.9 | 1.6 | Not measured | Not measured | Not measured |
| High-P (batch 2) | 129.5 | 49.8 | Not measured | Not measured | Not measured |
| Low-P (batch 3) | 9.9 | 1.9 | Not measured | Not measured | Not measured |
| High-P (batch 3) | 101.8 | 43.5 | Not measured | Not measured | Not measured |

**Table S2: Marginal ANOVA analysis to determine the impact of media and phage infection on each biomolecule type.** Both media (high-P versus low-P) and phage infection (infected versus uninfected) have significant impacts on all omics data types, as indicated by *P* values<0.01. Higher Chi^2^ values represent greater relative importance of the environment (i.e. being in high-P versus low-P) or the infection.

| **Data type** | **Chi^2^ value, influence of:** | | ***P* values,**  marginal ANOVA |  |
| --- | --- | --- | --- | --- |
|  | *Environment* | *Phage infection* |  |  |
|  |  |  |  |  |
| Phage Transcriptomes | 78 | 21 | <0.0001, media and phage |  |
| Phage Proteomes | 1690 | 26 | <0.0001, media and phage |  |
| Virocell Transcriptomes | 70,600 | 191,000 | <0.0001, media and phage |  |
| Virocell Proteomes | 3990 | 4000 | <0.0001, media and phage |  |
| Virocell Endo-metabolomes | 213 | 70 | <0.0001, media and phage |  |
| Virocell Exo-metabolomes | 2250 | 2132 | <0.0001, media and phage |  |
| Virocell Endo-lipidomes | 269 | 2670 | <0.0001, media and phage |  |

**Table S3: Linear mixed effects models of the transcriptomics data.** A marginal ANOVA analysis on the (i) overall differences of transcriptomics counts (log_2_FPKM), (ii) environment-specific differences in transcriptomics counts (log_2_FPKM) for each treatment, and (iii) treatment-specific differences (one phage versus the other) in transcriptomics counts (log_2_FPKM) in each of the high-P and low-P conditions. The *P* values indicate if a variable significantly affects the response variable after controlling for other variables. The relative importance of the predictors can be inferred from the chi-squared values. “Infection type” indicates HP1-virocell vs HS2-virocell, “Media type” indicates high-P versus low-P, “Time” indicates time points collected during infection, “Control” indicates the uninfected host.


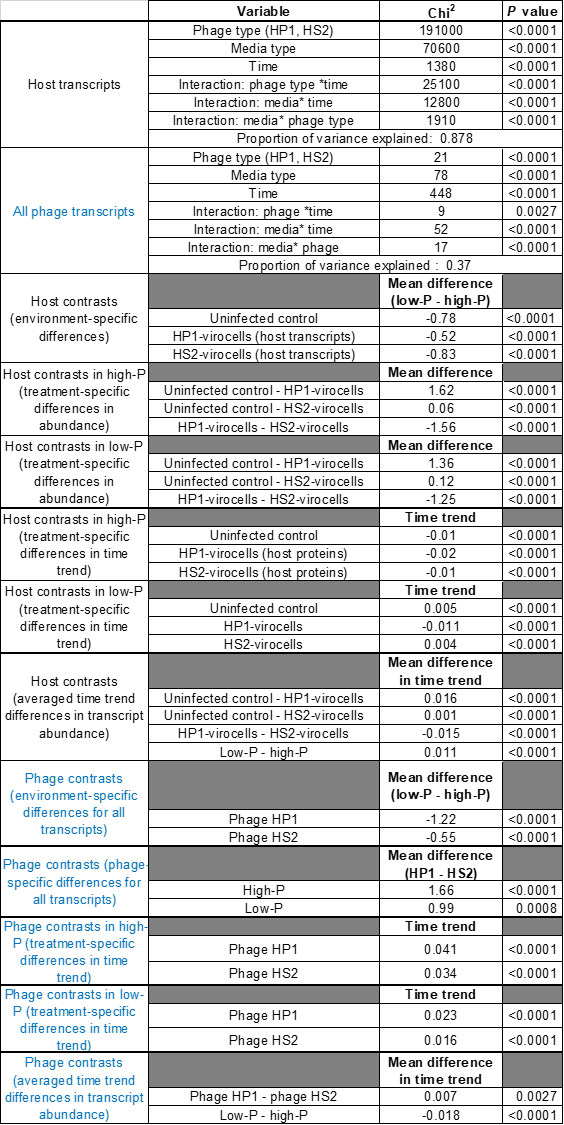


**Table S4: Linear mixed effects model results on the proteomics data.** A marginal ANOVA analysis on the (i) overall differences of proteome counts, (ii) environment-specific differences in proteome counts for each treatment, and (iii) treatment-specific differences (one phage versus the other) in proteome counts in each of the high-P and low-P conditions. The *P* values indicate if a variable significantly affects the response variable after controlling for other variables. The relative importance of the predictors can be inferred from the chi-squared values. “Infection type” indicates HP1-virocell vs HS2-virocell, “Media type” indicates high-P versus low-P, “Time” indicates time points collected during infection, “Control” indicates the uninfected host.

**
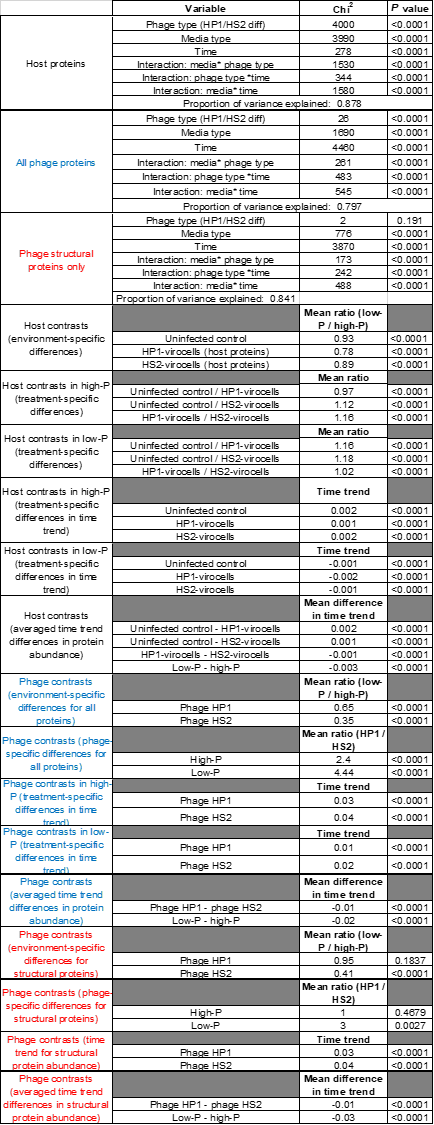
**

**Table S5: Linear mixed effects model results from the intracellular lipidomics data.** A marginal ANOVA analysis on the (i) overall differences of intensities in the virocell lipidome, (ii) environment-specific differences in lipid intensities for each treatment, and (iii) treatment-specific differences (one phage versus the other) in lipid intensities in each of the high-P and low-P conditions. The *P* values indicate if a variable significantly affects the response variable after controlling for other variables. The relative importance of the predictors can be inferred from the chi-squared values. “Infection type” indicates HP1-virocell vs HS2-virocell, “Media type” indicates high-P versus low-P, “Time” indicates time points collected during infection, “Control” indicates the uninfected host.


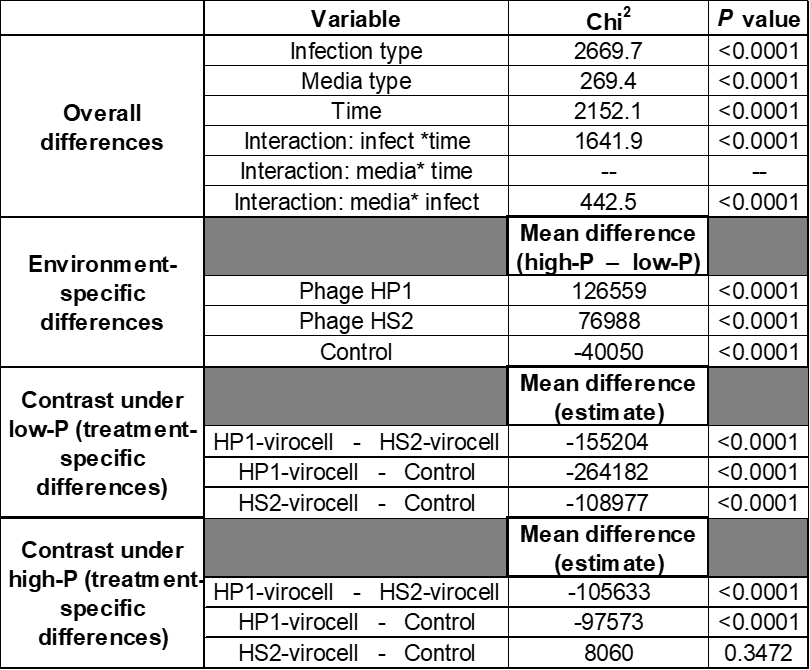


**Table S6: Dissolved organic carbon (DOC) chemical compound statistics.** Shown are the statistical results for DOC chemical compounds by i) DOC class, ii) Gibbs free energy, iii) Rao’s quadratic entropy - elemental composition, iv) network heterogeneity, and v) Rao’s quadratic entropy - reactivity. The tests compare all cells (uninfected control cells, HP1-virocells, HS2-virocells) in high-P versus low-P conditions, or each cell against each other in each condition (e.g. uninfected control cells versus HS2-virocells in low-P conditions).


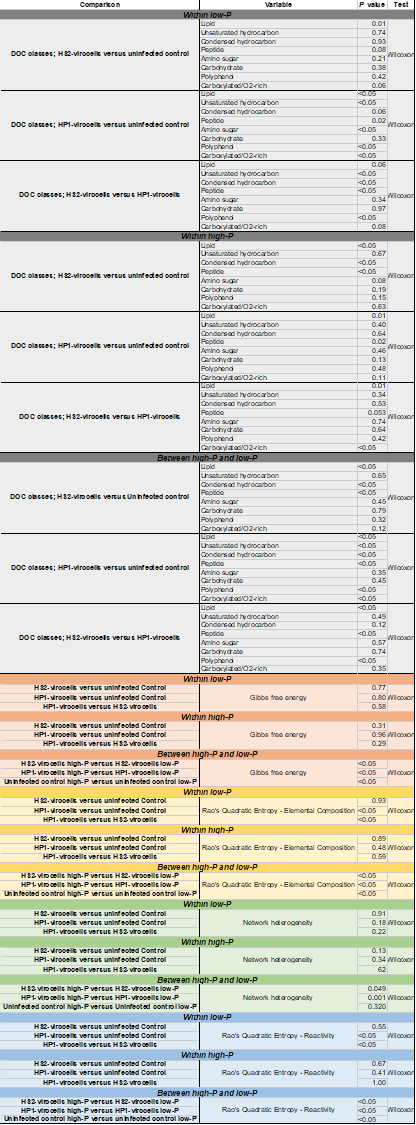


# **Supplementary references:**

1. Howard-Varona C, Lindback MM, Bastien GE, Solonenko N, Zayed AA, Jang HB, et al. Phage-specific metabolic reprogramming of virocells. *ISME J*. 2020;14(4):881–95.

2. Howard-Varona C, Roux S, Dore H, Solonenko NE, Holmfeldt K, Markillie LM, et al. Regulation of infection efficiency in a globally abundant marine *Bacteriodetes* virus. *ISME J*. 2017;11(1):284–95.

3. Howard-Varona C, Hargreaves KR, Solonenko NE, Markillie LM, White RA, Brewer HM, et al. Multiple mechanisms drive phage infection efficiency in nearly identical hosts. *ISME J*. 2018;12(6):1605–18.

4. Howard-Varona C, Roux S, Bowen BP, Silva LP, Lau R, Schwenck SM, et al. Protist impacts on marine cyanovirocell metabolism. *ISME Commun*. 2022;2(1):1–14.

5. Robinson MD, McCarthy DJ, Smyth GK. edgeR: a Bioconductor package for differential expression analysis of digital gene expression data. *Bioinformatics*. 2010;26(1):139–40.

6. Mortazavi A, Williams BA, McCue K, Schaeffer L, Wold B. Mapping and quantifying mammalian transcriptomes by RNA-Seq. *Nature Methods*. 2008;5(7):621–8.

7. Langfelder P, Zhang B, Horvath S. Defining clusters from a hierarchical cluster tree: the dynamic tree cut package for R. *Bioinformatics*. 2007;24(5):719–20.

8. Folch J, Lees M, Sloane Stanley GH. A simple method for the isolation and purification of total lipides from animal tissues. *J Biol Chem*. 1957;226(1):497–509.

9. Nakayasu ES, Nicora CD, Sims AC, Burnum-Johnson KE, Kim YM, Kyle JE, et al. MPLEx: a robust and universal protocol for single-sample integrative proteomic, metabolomic, and lipidomic analyses. *mSystems*. 2016;1(3):10.1128/msystems.00043-16.

10. Kyle JE, Crowell KL, Casey CP, Fujimoto GM, Kim S, Dautel SE, et al. LIQUID: an-open source software for identifying lipids in LC-MS/MS-based lipidomics data. *Bioinformatics*. 2017;33(11):1744–6.

11. Pluskal T, Castillo S, Villar-Briones A, Orešič M. MZmine 2: Modular framework for processing, visualizing, and analyzing mass spectrometry-based molecular profile data. *BMC Bioinformatics*. 2010;11(1):395.

12. Snijders AM, Langley SA, Kim YM, Brislawn CJ, Noecker C, Zink EM, et al. Influence of early life exposure, host genetics and diet on the mouse gut microbiome and metabolome. *Nat Microbiol*. 2016;2(2):1–8.

13. Hiller K, Hangebrauk J, Jäger C, Spura J, Schreiber K, Schomburg D. MetaboliteDetector: comprehensive analysis tool for targeted and nontargeted GC/MS based metabolome analysis. *Anal Chem*. 2009;81(9):3429–39.

14. Dittmar T, Koch B, Hertkorn N, Kattner G. A simple and efficient method for the solid-phase extraction of dissolved organic matter (SPE-DOM) from seawater. *Limnology and Oceanography*: Methods. 2008;6(6):230.

15. Pang Z, Chong J, Zhou G, de Lima Morais DA, Chang L, Barrette M, et al. MetaboAnalyst 5.0: narrowing the gap between raw spectra and functional insights. *Nucleic Acids Research*. 2021;49(W1):W388–96.

16. Tolić N, Liu Y, Liyu A, Shen Y, Tfaily MM, Kujawinski EB, et al. Formularity: software for automated formula assignment of natural and other organic matter from ultrahigh-resolution mass spectra. *Anal Chem*. 2017;89(23):12659–65.

17. Tfaily MM, Chu RK, Toyoda J, Tolić N, Robinson EW, Paša-Tolić L, et al. Sequential extraction protocol for organic matter from soils and sediments using high resolution mass spectrometry. *Anal Chim Acta*. 2017;972:54–61.

18. Ayala-Ortiz C, Graf-Grachet N, Freire-Zapata V, Fudyma J, Hildebrand G, AminiTabrizi R, et al. MetaboDirect: an analytical pipeline for the processing of FT-ICR MS-based metabolomic data. *Microbiome*. 2023;11(1):28.

19. Burgess KEV, Borutzki Y, Rankin N, Daly R, Jourdan F. MetaNetter 2: A Cytoscape plugin for ab initio network analysis and metabolite feature classification. *J Chromatogr B Analyt Technol Biomed Life Sci*. 2017;1071:68–74.

20. Longnecker K, Kujawinski EB. Using network analysis to discern compositional patterns in ultrahigh-resolution mass spectrometry data of dissolved organic matter. *Rapid Commun Mass Spectro*m. 2016;30(22):2388–94.

21. Flamholz A, Noor E, Bar-Even A, Liebermeister W, Milo R. Glycolytic strategy as a tradeoff between energy yield and protein cost. *Proc Natl Acad Sci USA*. 2013;110(24):10039–44.
